# Near-infrared photoimmunotherapy for effective elimination of ovarian cancer cells by inducing immunogenic cell death

**DOI:** 10.1016/j.omton.2025.201086

**Published:** 2025-11-12

**Authors:** T.M. Mohiuddin, Chaoyu Zhang, Wenjie Sheng, Marwah Al-Rawe, Roland Schmitz, Marcus Niebert, Natalia El-Merhie, Felix Zeppernick, Ivo Meinhold-Heerlein, Ahmad Fawzi Hussain

**Affiliations:** 1 Department of Gynecology and Obstetrics, Medical Faculty, Justus-Liebig-University Giessen, Klinikstr. 33, 35392 Giessen, Germany; 2 Clinic for Gynecology and Obstetrics, University Hospital Brandenburg, Medizinische Hochschule Brandenburg Campus GmbH, Hochstraße 29, 14770 Brandenburg an der Havel, Germany; 3 Institute of Pathology, University Hospital Giessen, Justus-Liebig-University Giessen, Langhanssstr. 10, 35392 Giessen, Germany; 4 Institute for Lung Health (ILH), Cardiopulmonary Institute (CPI), Member of the German Center for Lung Research (DZL), Justus Liebig University Giessen, Aulweg 128, 35392 Giessen, Germany

**Keywords:** near-infrared photoimmunotherapy, ovarian cancer, cancer antigen, scFv, SNAP tag, IR700, immunogenic cell death, dendritic cell maturation

## Abstract

Ovarian cancer represents one of the most common forms of female gynecological cancer. The existing treatment modalities include surgery and chemotherapy, which are unable to treat local and long-distant micro-metastases. Near-infrared photoimmunotherapy (NIR-PIT) is a newly developed treatment strategy that can selectively kill the cancer cells and induce immunogenic cell death and thereby stimulate anti-tumor immune responses. In this study, we developed five NIR-PIT agents by conjugating scFv-SNAP-tag fusion proteins with BG-modified IRdye700 to target ovarian cancer cells, which expressed cell surface antigens epidermal growth factor receptor (EGFR), Her2, FOLR1, TROP2, and TF. Flow cytometry and microscopic studies confirmed the specificity of all the investigated NIR-PIT agents binding to corresponding overexpressed cancer cells. In addition, all the investigated NIR-PIT agents demonstrate specific binding to human ovarian cancer tissues, as confirmed by multiplex immunofluorescence imaging. We validated that all five NIR-PIT agents decreased the cell viability in a concentration-dependent manner with IC_50_ values of ∼42–283 nM. Moreover, all examined NIR-PIT agents induced cell death with efficiencies of ∼80%–92%. The NIR-PIT agents triggered major hallmarks of immunogenic cell death like cell surface expression of calreticulin, HSP70, and HSP90 and the extracellular release of ATP and HMGB1. Furthermore, co-culturing immature dendritic cells with dying cancer cells targeted by EGFR and TF NIR-PIT agents mediated enhanced dendritic cell maturation, as indicated by increased expression of CD80, CD86, CD40, and HLADR. Taken together, our results suggested that all five investigated NIR-PIT agents have great potential to be applicable for ovarian cancer treatment.

## Graphical abstract

**Figure:**
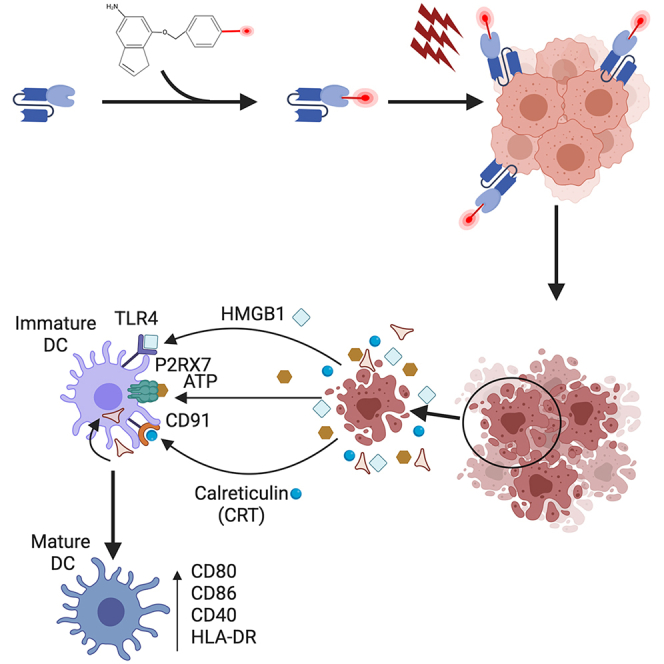

## Teaser

Five cancer antigen-specific NIR-PIT agents targeting EGFR, Her2, FOLR1, TROP2, and TF were developed to selectively kill ovarian cancer cells. These agents induced immunogenic cell death and promoted dendritic cell maturation, demonstrating strong potential for immune-activating ovarian cancer therapy.

## Introduction

Ovarian cancer is the most fatal female gynecological cancer.^1^ Rapid disease development (local to distance metastasis), resistance to therapies, and relapse after treatment are also responsible for the high mortality rate of ovarian cancer.^2^ Debulking surgery is the most commonly used treatment for ovarian cancer patients by which maximal reduction of tumor and precise intra-abdominal staging can be possible.^3^ For stage III–IV ovarian cancer patients, primary debulking surgery followed by platinum-based chemotherapy has become the standard treatment.^4^^,^^5^ In most of the patients, relapse occurs following surgery and chemotherapy, highlighting the need to develop new therapeutic strategies that could reduce the side effects and residual tumor.^6^

Molecular targeted therapies are emerging as ground-breaking and promising cancer treatment strategies that inhibit cancer growth and metastasis by interfering with specific molecules. In gynecological cancer, potential targeted therapeutic agents include poly ADP-ribose polymerase (PARP) inhibitors, angiogenesis inhibitors, inhibitors for DNA repair mechanisms, tumor-intrinsic signaling pathway inhibitors, and selective estrogen receptor down-regulators.^7^^,^^8^ Moreover, some PARP inhibitors (olaparib, rucaparib, niraparib) have been approved by US Food and Drug Administration for the treatment of advanced or recurrent ovarian cancer.^8^

Near-infrared photoimmunotherapy (NIR-PIT) is a newly developed cancer treatment approach that utilizes monoclonal antibodies (mAbs) or their fragments conjugated with a photo-activable phthalocyanine-derivative dye, IRDye700DX (IR700).^9^^,^^10^^,^^11^ First, the antibody IR700 conjugates is introduced into the patient to bind to the cancer cells. After local exposure of NIR light to the tumor, rapid and selective cancer cell death occurred by activating the antibody IR700 conjugates.^12^^,^^13^^,^^14^ The mechanisms of NIR-PIT-induced cell death remain unclear. It is reported that the photochemical reaction changes the antibody-IR700 conjugate-bound cell membrane to water-insoluble aggregates that causes cell swelling and subsequently releases the intracellular materials, although leading to necrotic cell death.^9^^,^^10^^,^^15^^,^^16^^,^^17^ This rapid cell lysis leads to the release of cytoplasmic antigens and damage-associated molecular patterns (DAMPs) such as cell-surface exposed calreticulin, high-mobility group box 1 (HMGB1), adenosine triphosphate (ATP), heat shock protein (HSP) 70, and HSP90, which is a characteristic feature of immunogenic cell death (ICD).^9^^,^^11^^,^^18^^,^^19^^,^^20^ These DAMP markers are responsible for the maturation of dendritic cells (DCs) that prime the naive CD8^+^ T cells, which then proliferate due to the rapid release of multiple neoantigens and can attack the residual cancer cells. The induced immune cells can enhance systemic anticancer immune response via their migration throughout the body and by attacking distant metastatic sites.^12^

Many NIR-PIT agents were developed for targeting different types of cancers in the last decade. At the beginning, epidermal growth factor receptor (EGFR)- and human epidermal growth factor receptor 2 (Her2)-targeted NIR-PIT agents were developed and then expanded to target diverse cancer cell surface antigens such as epithelial cell adhesion molecule (EpCAM), program death ligand 1 (PD-L1), prostate-specific membrane antigen (PMSA), cluster of differentiation (CD)44, CD47, CD20, CD25, and CD29 by using mAb and their fragments.^13^ The target selection for the development of NIR-PIT depends on the overexpression of certain receptors on the cell surface of cancer cells. Several cell surface receptors were identified in ovarian cancer based on expression profile analysis, which led to the development of NIR-PIT.^21^^,^^22^ EGFR,^23^^,^^24^ Her2,^25^ folate receptor α (FOLR1),^26^ trophoblast cell surface antigen 2 (TROP2),^27^^,^^28^^,^^29^^,^^30^ and tissue factor (TF)^31^^,^^32^ are overexpressed in ovarian cancer and play crucial roles in epithelial tumor growth, invasion, and metastasis, which makes them attractive targets for diagnosis and therapy of ovarian cancers.

In NIR-PIT, mAb and antibody derivatives, such as fragment antigen-binding (Fab), single-chain antibody fragment (scFv), affibody, diabody, minibody, and nanobody, as well as antibody mimetics were used to conjugate IR700 for producing photo-immunoconjugates.^11^^,^^32^^,^^33^^,^^34^^,^^35^ Compared to mAbs, scFvs provide several advantages, as they retain the complete antigen-binding capability,^36^ while simultaneously having better tumor penetration due to their small size, reduced immunogenicity, lower retention times in nontarget tissue, and more rapid blood clearance.^37^^,^^38^^,^^39^ scFv is extensively used to develop several molecular-targeted therapies including targeted photodynamic therapy, antibody-drug conjugates, or photoimmunotherapy.^40^^,^^41^^,^^42^ SNAP-tag is a promising site-specific conjugation method that reacts with benzylguanine (BG) derivatives.^43^^,^^44^ SNAP-tag-based antibody fusion protein is used to couple BG-modified auristatin F in an irreversible 1:1 stoichiometric reaction.^42^^,^^45^^,^^46^ In this study, we employed SNAP-tag technology to generate scFv-SNAP-IR700 fusion proteins for NIR-PIT. Compared with conventional mAb-IR700 conjugates, the SNAP-tag system provides site-specific and stoichiometric conjugation, ensuring uniform labeling and preserving antigen-binding activity.^35^^,^^40^^,^^41^^,^^47^

In this study, we have developed five NIR-PIT agents by conjugating IR700 to scFvs targeting EGFR, Her2, FOLR1, TROP2 and TF using SNAP-tag technology. We evaluated the binding specificities of NIR-PIT agents in human ovarian cancer tissues by tyramide signal amplification (TSA)-based multiplex immunofluorescence (mIF) analysis. *In vitro* NIR-PIT agents were validated by investigating the specificity and cell viability. We demonstrated that NIR-PIT induced necrosis cell death and triggered release of ICD hallmarks (calreticulin, HSP70, HSP90, ATP, and HMGB1), which subsequently induced DC maturation *in vitro*. Both *in vitro* and *ex vivo* results revealed that our developed NIR-PIT agents hold substantial promise for targeted ovarian cancer therapy, as they combine high specificity, adaptability, and precise control over treatment areas.

## Results

### Ovarian cancer cells express different level of cell surface antigens

**Figure 1:**
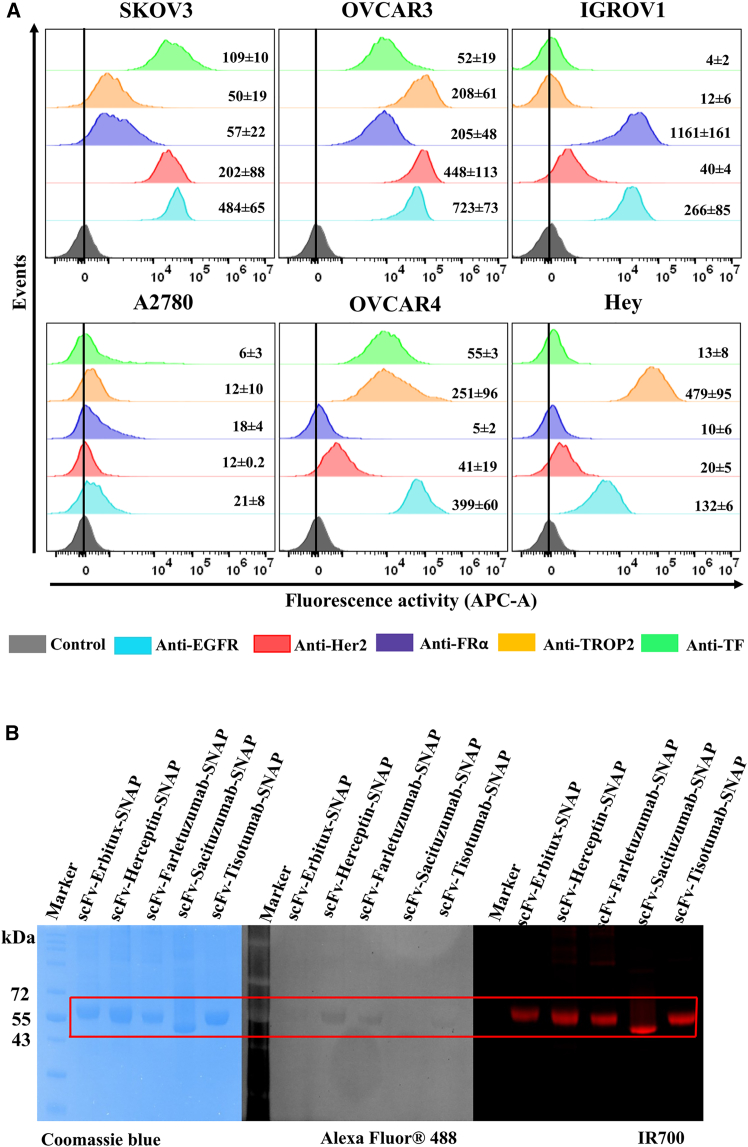
Expression pattern of five tumor-associated antigens (EGFR, Her2, FOLR1, TROP2 and TF) in six ovarian cancer cell lines and SNAP-tag proteins labeled with BG-IR700 (A) The antigen expression in ovarian cancer cell lines was determined by flow cytometry. Cells were treated with anti-EGFR (EGFR mAb, H11), anti-Her2 (ErbB2 mAb, 3B5), anti FOLR1 (FOLR1 mAb, 548908), anti-TROP2 (TROP2 mAb, MR54), and anti TF (CD142 mAb, HTF-1) antibodies, followed by incubation with the secondary antibody (goat anti-mouse IgG highly cross-adsorbed secondary antibody, Alexa Fluor Plus 647). The ΔMFI values are shown in each histogram (mean ± SE) from three independent experiments. The ΔMFI was calculated from the MFI of the cells expressing the marker of interest divided by the MFI of the unstained cells. (B) Coomassie blue staining represents IR700-conjugated scFv-Erbitux-SNAP, scFv-Herceptin-SNAP, scFv-Farletuzumab-SNAP, scFv-Sacituzumab-SNAP, and scFv-Tisotumab-SNAP followed by post-incubation with SNAP-Surface Alexa Fluor 488. Corresponding Alexa Fluor 488 fluorescence and IR700 signals visualized with gel documentation system (Bio-Rad) and Odyssey DLx Imager, respectively. Broad range marker, 11–250 kDa. The red box indicates the corresponding protein bands.

**Figure 2:**
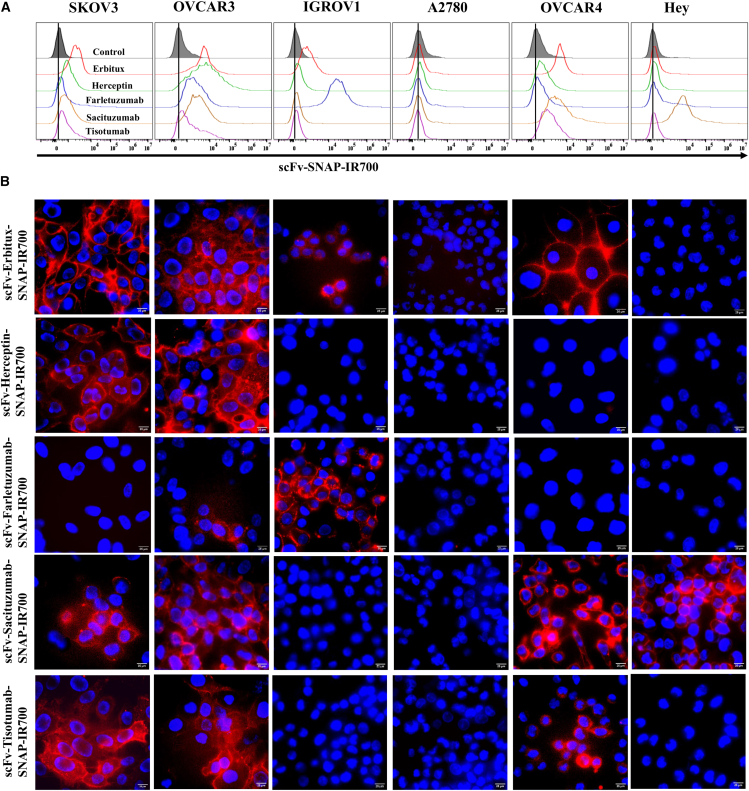
NIR-PIT agents specifically bind to ovarian cancer cells (A) Specific binding of NIR-PIT agents to ovarian cancer cell lines was analyzed by flow cytometry. The flow cytometric histograms represent the binding of scFv-Erbitux-SNAP-IR700, scFv-Herceptin-SNAP-IR700, scFv-Farletuzumab-SNAP-IR700, scFv-Sacituzumab-SNAP-IR700, and scFv-Tisotumab-SNAP-IR700 to EGFR^+^, Her2^+^, FOLR1^+^, TROP2^+^, and TF^+^ ovarian cancer cells. (B) Specific binding was also analyzed using fluorescence microscopy. Fluorescence images were obtained for the EGFR^+^, Her2^+^, FOLR1^+^, TROP2^+^, and TF^+^ cells. The DAPI and IR700 fluorescence signals are overlaid to visualize their binding specificities.

The flow cytometric analysis showed strong binding signal of anti-EGFR-647 in SKOV3, OVCAR3, IGROV1, OVCAR4, and Hey cell lines. The average difference in fluorescence intensity between stained and unstained cells (delta mean fluorescence intensity [ΔMFI]) was used as an indicator of antigen expression. The ΔMFI values were ∼484, ∼723, ∼266, ∼399 and ∼132 in SKOV3, OVCAR3, IGROV1, OVCAR4, and Hey cells respectively, representing higher expression of EGFR. In contrast, A2780 cells express low levels of EGFR (Figure 1A). Similarly, SKOV3 and OVCAR3 cells showed high fluorescence signals, when staining with anti-Her2-647. The ΔMFI values were ∼202 and ∼448 in SKOV3 and OVCAR3 cells, respectively, whereas IGROV1, A2780, OVCAR4, and Hey cell lines exhibited low shift of fluorescence signals with ΔMFI values of ∼40, ∼12, ∼41 and ∼20 respectively. As shown in Figure 2, the ΔMFI values of FOLR1 in OVCAR3 and IGROV1 cells were ∼205 and ∼1,161 whereas the other four cell lines (SKOV3, A2780, OVCAR4, and Hey) showed low to moderate shift of fluorescence signals. The ΔMFI values of FOLR1 were ∼57, ∼18, ∼5, and ∼10 in SKOV3, A2780, OVCAR4, and Hey cells, respectively. These results suggest that OVCAR3 and IGROV1 cells expressed high level of FOLR1. In addition, OVCAR3 (ΔMFI∼208), OVCAR4 (ΔMFI∼251), and Hey (ΔMFI∼476) cells expressed high levels of TROP2, whereas SKOV3 (ΔMFI∼50) cells expressed moderate level of TROP2. On the other hand, IGROV1 and A2780 cells showed low level of TROP2, and their ΔMFI was ∼12 for both cell lines. Moreover, the TF was highly expressed in SKOV3 cells and ΔMFI was ∼109. OVCAR3 (ΔMFI∼52) and OVCAR4 (ΔMFI∼55) cells exhibited moderate level of TF. On the contrary, IGROV1, A2780, and Hey cells showed low level of TF and their ΔMFI was less than 14 (Figure 1A).

### SNAP-tag generates functional scFv-SNAP-based NIR-PIT agents

To site-specifically conjugate IR700 to scFv-SNAP-tag fusion protein (Figures S1 and S2), IR700 molecules were modified with BG using BG-PEG-NH_2_ linkers. The chemical coupling and purification of IR700 with BG linkers was confirmed by high-performance liquid chromatography (HPLC) and mass spectrometry analysis (Figure S3). The labeling efficiency of scFv-SNAP-tag fusion proteins was further determined by coupling them to BG-IR700. The coupling efficiency of BG-IR700 to the fusion proteins was confirmed by visualizing the IR700 signal at 700 nm using an Odyssey DLx Imager (LI-COR Bioscience) (Figure 1B). The conjugation was further confirmed by post-incubating them with SNAP-Surface Alexa Fluor 488 (New England Biolabs). As shown in Figure 1B, the specific coupling site was blocked by BG-IR700, which inhibited the coupling with SNAP-Surface Alexa Fluor 488. This result confirms that BG-IR700 coupled site specifically, irreversibly to SNAP-tag fusion proteins within 2 h at room temperature.

### Specific binding of NIR-PIT agents on ovarian cancer cells

To analyze the binding specificities of five scFv-SNAP-tag proteins, IR700 was conjugated with fusion protein and analyzed by flow cytometry and fluorescence microscopy. The flow cytometry and microscopic data revealed that scFv-Erbitux-SNAP-IR700 specifically bound to the EGFR high-expressing SKOV3, OVCAR3, and OVCAR4 cells, while only a minimal fluorescence shift was observed on the EGFR low-expressing A2780 cells (Figure 2A). Similarly, strong binding signal was observed in Her2 high-expressing SKOV3 and OVCAR3 cell lines after incubation with scFv-Herceptin-SNAP-IR700 (Figure 2). After incubation of ovarian cancer cells with scFv-Farletuzumab-SNAP-IR700, high-FOLR1-expressing IGROV1 cells exhibited strong binding and moderate-FOLR1-expressing OVCAR3 cells showed moderate binding, whereas no fluorescence signal was detected in low-FOLR1-expressing cell lines (Figure 2). In addition, we observed enhanced binding of scFv-Sacituzumab-SNAP-IR700 to SKOV3, OVCAR3, OVCAR4, and Hey cells compared to IGROV1 and A2780 cells (Figure 2). Moreover, the scFv-Tisotumab-SNAP-IR700 showed enhanced binding to SKOV3, OVCAR3, and OVCAR4 cells relative to IGROV1, A2780, and Hey cells (Figure 2; Table S4).

### Evaluating the expression pattern and binding specificities of NIR-PIT agents to ovarian cancer tissues

**Figure 3:**
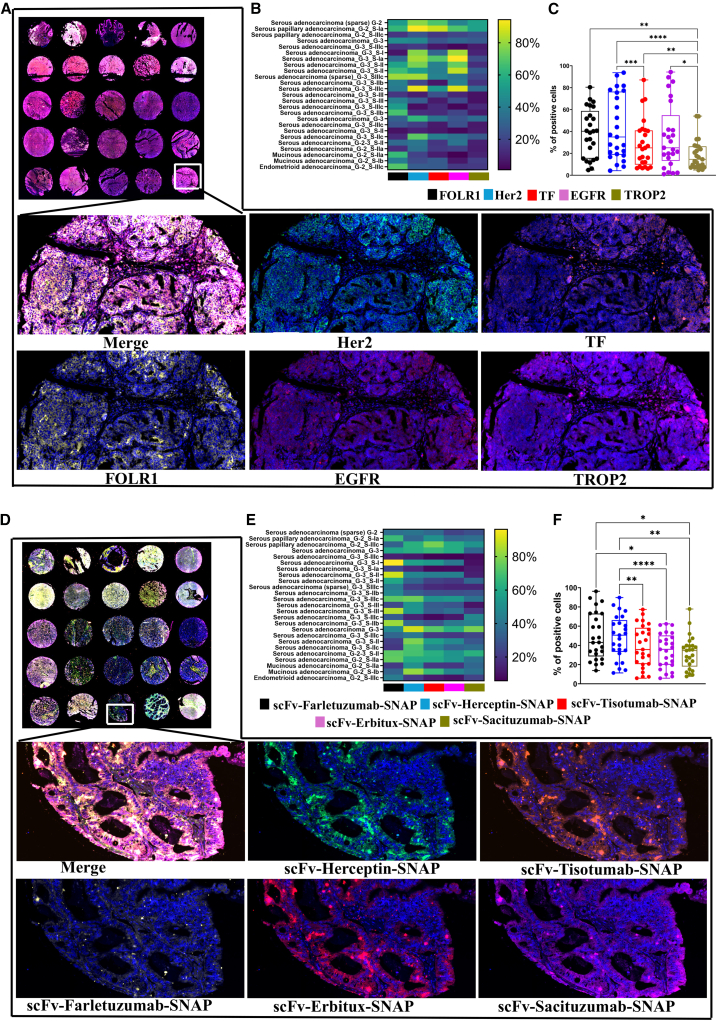
Expression profiles of EGFR, Her2, FOLR1, TROP2, and TF and binding of NIR-PIT agents to the ovarian cancer tissues (A) Microphotographs of mIF of EGFR, Her2, FOLR1, TROP2, and TF antigens in 25 different ovarian cancer tissues along with corresponding 100x sample regions from one core for each marker from five different channels. (B) Heatmap shows the expression pattern of EGFR, Her2, FOLR1, TROP2, and TF in 25 ovarian cancer tissue cores. (C) The boxplot represents the average percentages of each marker in 25 ovarian cancer tissue cores. (D) Microphotographs of mIF of binding specificities of five NIR-PIT agents in 25 different ovarian cancer tissues along with corresponding 100x sample regions from one core for each NIR-PIT agent in five different channels. (E) The percentage of each NIR-PIT agent in 25 cores is shown in heatmap. (F) The average percentage of each NIR-PIT agent binding is described in boxplot with error bar (mean ± SD). The statistical analysis was carried out using one-way ANOVA and “mvt” adjustment for Figure 3C and 3F. Figure 3C and 3F contain ∗*p* ≤ 0.05, ∗∗*p* ≤ 0.01, ∗∗∗*p* ≤ 0.001, ∗∗∗∗*p* ≤ 0.0001.

To illustrate the expression pattern of cancer markers in ovarian cancer tissues, TSA-based mIF method was applied to simultaneously determine the expression pattern of five cancer markers in tissue microarray containing 25 cases of ovarian carcinoma (22 serous adenocarcinomas, 2 mucinous adenocarcinomas, and 1 endometrioid adenocarcinoma). Figure 3A shows cell surface expressions of EGFR, Her2, FOLR1, TROP2, and TF in 25 different ovarian cancer tissues (OV1003, TissueArray.com), and the expressions were observed predominantly on the cell membranes of ovarian cancer glands. To simultaneously visualize the expression patterns of cancer cell surface antigens in different ovarian cancer tissues, the percentage of positive cells from mIF are shown in heatmap. Serous adenocarcinoma tissues express high levels of EGFR and Her2. The mucinous and endometrioid adenocarcinoma tissues express moderate level of FOLR1. In addition, we demonstrated that the average percentages of FOLR1- and Her2-positive cells were significantly higher in all ovarian cancer tissues than TF and TROP2. To find out the clinical relevance of our NIR-PIT agents and the potential binding ability to ovarian cancer tissues, we also performed mIF using scFv-SNAP fusion proteins. Figure 3D shows that all investigated NIR-PIT agents bind to the ovarian cancer tissues in a pattern similar to that observed using commercial control antibodies in the multiplex panel (Figure 3A). The heatmap illustrates the relative densities of NIR-PIT agent-bound positive cells (Figure 3E). Interestingly, the average percentage of scFv-Farletuzumab-SNAP-binding cells was significantly higher than the scFv-Erbitux-SNAP and scFv-Sacituzumab-SNAP. In addition, scFv-Herceptin-SNAP had higher binding affinity to ovarian cancer tissues compared to the scFv-Erbitux-SNAP, scFv-Erbitux-SNAP, and scFv-Sacituzumab-SNAP (Figure 3F).

### NIR-PIT resulted in increased cell death in a concentration-dependent manner

**Figure 4:**
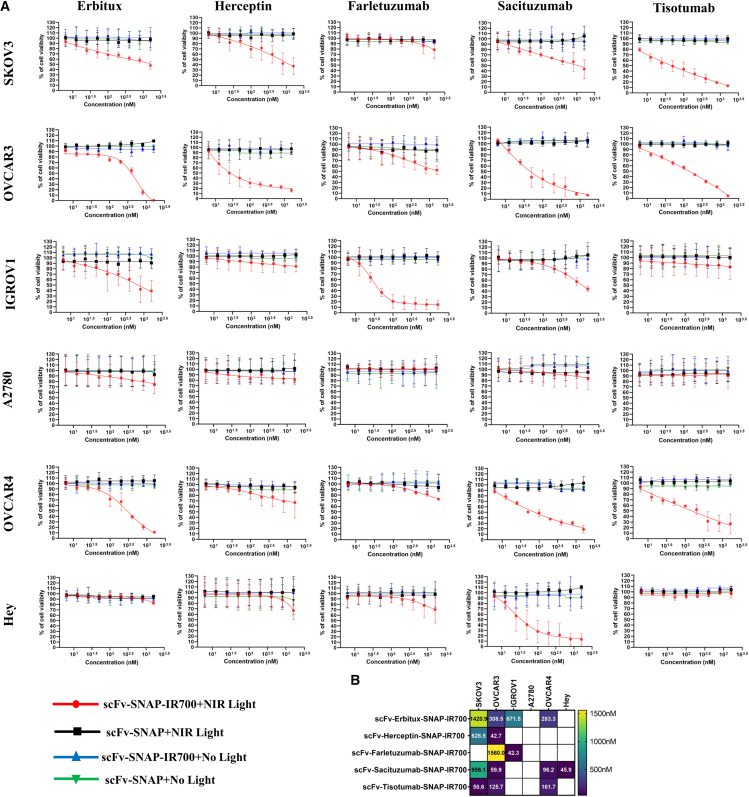
Evaluation of specific photocytotoxicity of five NIR-PIT agents in six ovarian cancer cells (A) Cells were treated with increasing concentration (6.25, 12.5, 25, 50, 100, 200, 400, 800, and 1,600 nM) of scFv-SNAP-IR700+NIR light (red), scFv-SNAP+NIR light (black), scFv-SNAP-IR700 (blue), and scFv-SNAP (green). Cell viability was determined by XTT Kit after 24-h treatment. (B) IC_50_ values of NIR-PIT agents are represented in the heatmap in which white block represents IC_50_ >1,600 nM. Data are shown in dose-response curve from triplicates of two independent experiments with error bar (mean ± SD).

As all NIR-PIT agents showed specific binding properties, it was hypothesized that they exhibited specific photocytotoxicity. Therefore, the photocytotoxic effect of scFv-SNAP-IR700-mediated NIR-PIT was evaluated in six ovarian cancer cell lines (SKOV3, OVCAR3, IGROV1, A2780, OVCAR4, and Hey). Cultured cells were incubated with different concentrations of scFv-Erbitux-SNAP-IR700, scFv-Herceptin-SNAP-IR700, scFv-Farletuzumab-SNAP-IR700, scFv-Tisotumab-SNAP-IR700, and scFv-Sacituzumab-SNAP-IR700 and then exposed to 690–710 nm (2 J/cm^2^) from a light-emitting diode (LED) light source. To measure cytotoxicity following NIR-PIT, XTT-based colorimetric cell proliferation assay was performed. After 24 h of light irradiation, high- to moderate-EGFR-expressing cell lines SKOV3, OVCAR3, IGROV1, and OVCAR4 cells treated with scFv-Erbitux-SNAP-IR700 showed increased cell death in a concentration-dependent manner. The IC_50_ values of SKOV3, OVCAR3, IGROV1, and OVCAR4 cells were 1,420, 308, 671, and 283 nM, respectively (Figure 4). The scFv-Erbitux-SNAP-IR700 showed negligible effects on both A2780 and Hey cell lines (Figure 4). Similarly, scFv-Herceptin-SNAP-IR700 showed significant cell death in high-Her2-overexpressed SKOV3 (IC_50_-628 nM) and OVCAR3 cells (IC_50_-42 nM). Percentage of dying cells was influenced by the concentration of scFv-Herceptin-SNAP-IR700. There were very little effects on Her2-low-expressed A2780, IGROV1, OVCAR4, and Hey cell lines. In addition, after treating the cells with scFv-Farletuzumab-SNAP-IR700, the cell viability was significantly reduced in high-FOLR1-expressing IGROV1 cells and moderate-FOLR1-expressing OVCAR3 cells. The IC_50_ values were 1,560 and 42 nM in OVCAR3 and IGROV1 cells, respectively. In addition, treating the cells with scFv-Sacituzumab-SNAP-IR700 decreased cell viability in high-TROP2-expressing OVCAR3, OVCAR4, and Hey cells and moderate-TROP2-expressing SKOV3 cells. The IC_50_ values were 59.9, 96.2, 45.9, and 956.1 nM in OVCAR3, OVCAR4, Hey, and SKOV3 cells, respectively. The scFv-Tisotumab-SNAP-IR700 killed high-TF-expressing cell lines SKOV3 (IC_50_: 50.6 nM), OVCAR3 (IC_50_: 125.7 nM), and OVCAR4 (IC_50_: 161.7 nM). There was no significant reduction of viability observed in any of the investigated cell lines without light activation or unconjugated scFv-SNAP (Figure 4).

### Receptor-mediated cell death induced by NIR-PIT agents

**Figure 5:**
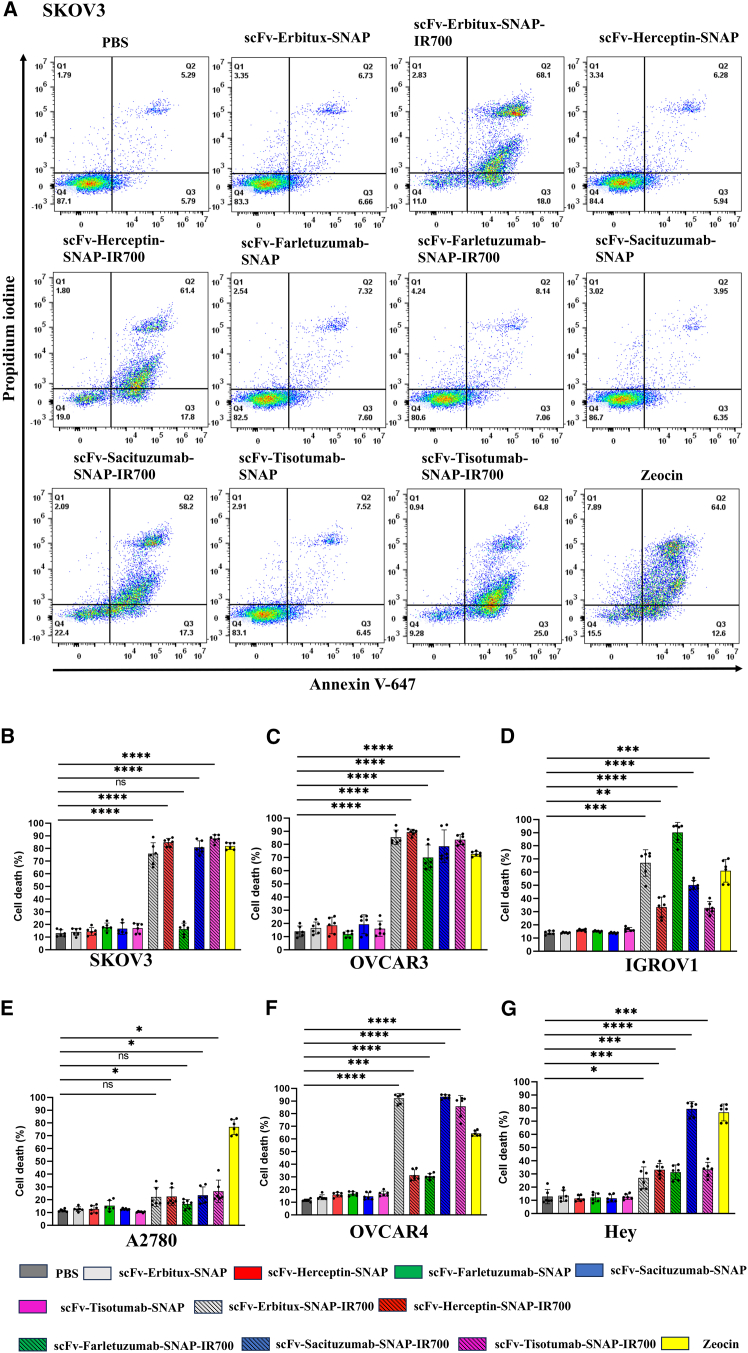
NIR-PIT agents induced cell death in SKOV3, OVCAR3, IGROV1, A2780, OVCAR4, and Hey cells. Cell death was measured by annexin assay after 24-h treatment (A) Flow cytometric data are shown in Scatterplot for SKOV3. (B–G) The cell death percentages are plotted in bar graph with mean ± SD from six biological replicates. Cells incubated with media or zeocin were used as negative or positive control. The statistical analysis was carried out using Welch one-way ANOVA and Dunnett’s test. ns, non-significant; ∗*p* ≤ 0.05; ∗∗*p* ≤ 0.01; ∗∗∗*p* ≤ 0.001; ∗∗∗∗*p* ≤ 0.0001.

The photocytotoxicity mediated by NIR-PIT agents was further validated by annexin assay following the protocol of Woitok et al.^48^ All six ovarian cell lines were treated with IR700-conjugated and -unconjugated five scFv-SNAP-tag fusion proteins. After 24 h of light irradiation, the percentage of cell death was measured. Negative and positive controls were set up by treating cells with PBS or zeocin, respectively. In SKOV3 cells, the scFv-Erbitux-SNAP-IR700, scFv-Herceptin-SNAP-IR700, scFv-Sacituzumab-SNAP-IR700, and scFv-Tisotumab-SNAP-IR700 induced significant programmed cell death with∼75%, ∼84%, ∼80%, and ∼87%, respectively, compared to untreated cells. In contrast, scFv-Farletuzumab-SNAP-IR700 treatment showed no or minimal increase in cell death (Figure 5). Similarly, all five NIR-PIT agents triggered significant level of cell death (∼70%–89%) in OVCAR3 cells, among which scFv-Herceptin-SNAP-IR700 showed ∼89% cell death. In IGROV1, scFv-Farletuzumab-SNAP-IR700 showed the highest toxicity (∼90%), while the rest of the NIR-PIT agents (scFv-Erbitux-SNAP-IR700, scFv-Herceptin-SNAP-IR700, scFv-Tisotumab-SNAP-IR700, and scFv-Sacituzumab-SNAP-IR700) also showed significant cell death (∼32%–67%) (Figures 5 and S4). In contrast, A2780 cells showed no significant effect after treatment7 with scFv-Erbitux-SNAP-IR700 (∼22%) and scFv-Farletuzumab-SNAP-IR700 (∼16%). On the other hand, scFv-Herceptin-SNAP-IR700, scFv-Tisotumab-SNAP-IR700, and scFv-Sacituzumab-SNAP-IR700 treatment causes ∼23%–26% cell death. Moreover, a significant increase (∼92%) in cell death was observed in OVCAR4 cells post-treatment with scFv-Erbitux-SNAP-IR700. In addition, scFv-Sacituzumab-SNAP-IR700- and scFv-Tisotumab-SNAP-IR700-treated OVCAR4 cells showed ∼92% and ∼85% cell death, respectively (Figures 5 and S5). The cell death was increased approximately 6-fold in Hey cells treated with scFv-Sacituzumab-SNAP-IR700 compared to the untreated cells. However, Hey cells treated with scFv-Erbitux-SNAP-IR700, scFv-Herceptin-SNAP-IR700, scFv-Farletuzumab-SNAP-IR700, and scFv-Tisotumab-SNAP-IR700 exhibited ∼27%–∼33% cell death (Figures 5 and S6).

### Determination of the type of cell death pathway triggered by NIR-PIT

**Figure 6:**
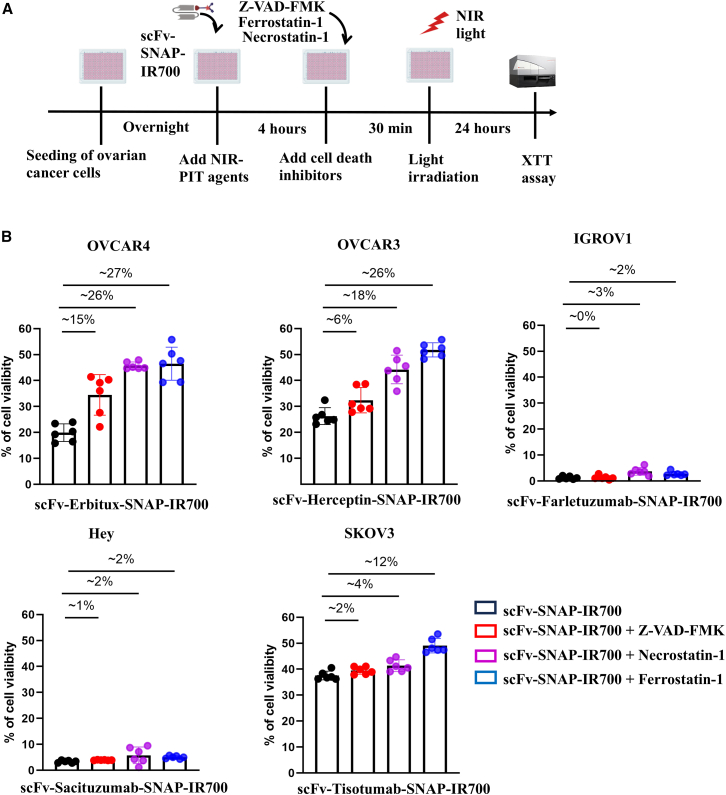
Determining the type of cell death pathway induced after NIR-PIT using cell death inhibitors (A) Schematic diagram of cell death pathway determination experiments. (B) Cell death measured by XTT assay after 24-h treatment. OVCAR4, OVCAR3, IGROV1, Hey, and SKOV3 were incubated with three different cell death inhibitors (z-VAD-FMK, Necrostatin-1, and Ferrostatin-1) followed by NIR-PIT. The cell viability is shown in bar graph with error bar (mean ± SD) from six biological replicates. The percentages of cell death rescuing are shown on the top of each bar and calculated by subtracting the percentage of cell viability of treatment from the control. Cells treated with scFv-SNAP-IR700 were used as a control.

To investigate the cell death pathway, we used three different cell death inhibitors including apoptosis inhibitor (z-VAD-FMK), necroptosis inhibitor (Necrostatin-1), and ferroptosis inhibitor (Ferrostatin-1). Following NIR-PIT, XTT assay was used after 24 h post-incubation. Cells without the cell death inhibitor were used as control. After treating OVCAR4 with scFv-Erbitux-SNAP-IR700, z-VAD-FMK, Necrostatin-1, and Ferrostatin-1 can rescue ∼15%, ∼26%, and ∼27% cell death, respectively (Figure 6). In addition, ∼26% cell death was rescued after adding Ferrostatin-1 and ∼18% cell death was inhibited after adding Necrostatin-1 in scFv-Herceptin-SNAP-IR700-treated OVCAR3 cells compared to the control. In contrast, there was no significant cell death inhibited after adding z-VAD-FMK in scFv-Herceptin-SNAP-IR700 treated OVCAR3 cells. As shown in Figure 6, z-VAD-FMK, Necrostatin-1, and Ferrostatin-1 could inhibit ∼5% cell death in scFv-Farletuzumab-SNAP-IR700-treated IGROV1. Similarly, the cell death was not significantly affected in Hey cells treated with scFv-Sacituzumab-SNAP-IR700 after adding z-VAD-FMK, Necrostatin-1, and Ferrostatin-1. Moreover, only Necrostatin-1 and Ferrostatin-1 partly inhibit scFv-Tisotumab-SNAP-IR700-induced cell death in SKOV3. In contrast, the z-VAD-FMK failed to rescue scFv-Tisotumab-SNAP-IR700-induced cell death in SKOV3 (Figure 6). These findings indicate that mixed types of cell death pathways are induced when treating with EGFR- and Her2-targeting NIR-PIT, including apoptosis, necroptosis, and ferroptosis. However, the inhibitors could not rescue high percentage of cell death after treating with FOLR1-, TROP2-, and TF-targeting NIR-PIT, indicating mixed cell death pathways are involved.

### NIR-PIT triggers ICD by the release of DAMPs

**Figure 7:**
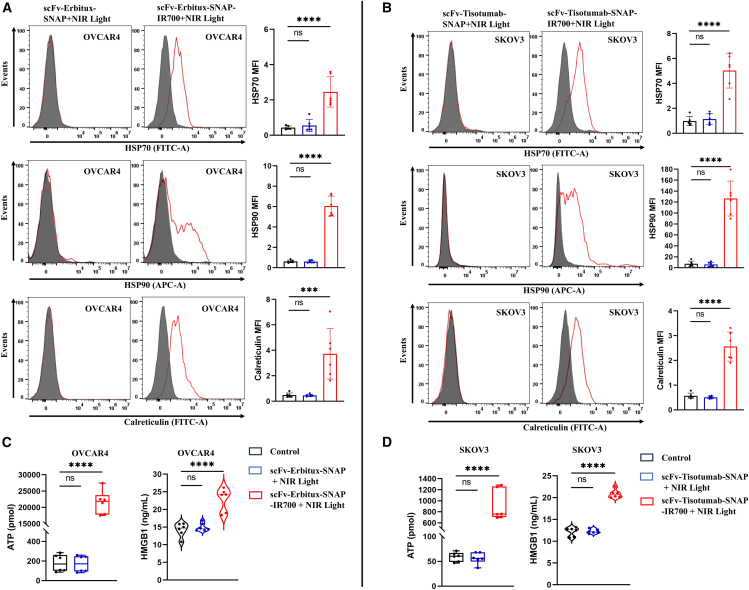
scFv-Erbitux-SNAP-IR700 and scFv-Tisotumab-SNAP-IR700 treatment triggers the release of ICD marker from OVCAR4 and SKOV3 (A) The flow cytometric histograms represent the cell surface expression of calreticulin (red), HSP70 (red), and HSP90 (red) and the control (filled gray) in scFv-Erbitux-SNAP-IR700-treated OVCAR4 cells and (B) scFv-Tisotumab-SNAP-IR700-treated SKOV3 cells. Only living cells were used in the analysis. MFI of cell surface calreticulin, HSP70, and HSP90 data are shown in bar graph with error bar (mean ± SD) from six biological replicates. (C) NIR-PIT induces a rapid release of HMGB1 and ATP from scFv-Erbitux-SNAP-IR700-treated OVCAR4 cells and (D) scFv-Tisotumab-SNAP-IR700-treated SKOV3 cells. After 24-h NIR light irradiation, extracellular ATP was measured by ATP luminescence assay and extracellular HMGB1 was measured by ELISA assay. Data are presented in boxplot (ATP release) and violin plot (HMGB1 release) as mean ± SD from six biological replicates. Cells exposed to NIR light irradiation without incubation with NIR-PIT agents were used as control. Statistical significance was determined by a one-way ANOVA and Dunnett’s test. ns, non-significant, ∗∗∗*p* ≤ 0.001, ∗∗∗∗*p* ≤ 0.0001.

As our investigated NIR-PIT agents kill the cancer cells by necrosis, we then investigated whether they could induce ICD. Calreticulin, HSP70, and HSP90 are expressed on the plasma membrane of cells undergoing ICD that facilitates DC maturation. Therefore, the major endogenous danger molecules calreticulin, HSP70, and HSP90 were analyzed using flow cytometry after 24 h post-irradiation. Irradiated cells without NIR-PIT agents were used as controls. OVCAR4 cells treated with scFv-Erbitux-SNAP-IR700 showed increased plasma membrane expression of calreticulin, HSP70, and HSP 90 compared to the control and cells treated with scFv-Erbitux-SNAP. In addition, the amounts of ATP (∼21 nmol) and HMGB1 (∼23 ng/mL) were significantly higher in scFv-Erbitux-SNAP-IR700-treated OVCAR4 cells compared to the control. In contrast, there were no significant differences of calreticulin, HSP70, and HSP90 expression and the extracellular release levels of ATP and HMGB1 in A2780 with both treatments (Figures 7 and S7). Similarly, scFv-Tisotumab-SNAP-IR700-treated SKOV3 cells resulted in enhanced cell surface expression of calreticulin, HSP70, and HSP90 compared to the control. The scFv-Tisotumab-SNAP-IR700-treated SKOV3 cells released ∼910 pmol of ATP and ∼21 ng/mL of HMGB1, whereas there was no significant difference of ATP and HMGB1 release in A2780 cells (Figures 7 and S7). In addition, scFv-Herceptin-SNAP-IR700-treated OVCAR3 cells, scFv-Farletuzumab-SNAP-IR700-treated IGROV1 cells, and scFv-Sacituzumab-SNAP-IR700-treated Hey cells exposed elevated levels of calreticulin, HSP70, and HSP90 on the plasma membrane compared to the control and released significant amounts of extracellular ATP and HMGB1 (Figures S8–S10).

### Maturation of DCs triggered by NIR-PIT-killed tumor cells

**Figure 8:**
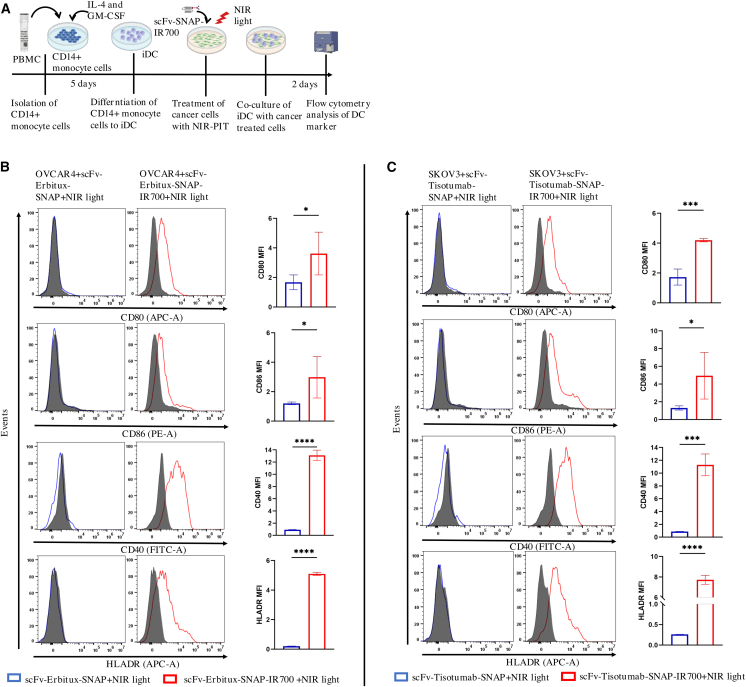
Co-culturing of NIR-PIT-treated ovarian cancer cells with iDCs promotes DC maturation (A) Schematic diagram of determining DC maturation experiments. (B) Immature DCs were cultured with scFv-Erbitux-SNAP-IR700-treated OVCAR4 cells or cultured with scFv-Erbitux-SNAP-treated OVCAR4 cells. (C) Immature DCs were cultured with scFv-Tisotumab-SNAP-IR700-treated SKOV3 cells or cultured with scFv-Tisotumab-SNAP-treated SKOV3 cells. After 48 h, the expressions of CD80, CD86, CD40, and HLADR on DCs were analyzed by flow cytometry. Immature DCs without co-culture are shown as a control. The expressions of CD80, CD86, HLADR, and CD40 on DCs are presented as flow cytometric histograms. The gray curves represent iDCs without co-culture. MFI of CD80, CD86, HLADR, and CD40 expression on DCs are shown in the bar graph. Graphs with error bars indicate mean ± SD from 3 biological replicates. Statistical significance was determined using unpaired *t* test. ∗*p* ≤ 0.05, ∗∗∗*p* ≤ 0.001, ∗∗∗∗*p* ≤ 0.0001.

Next, we examined whether our investigated NIR-PIT-killed cells can induce DC maturation *in vitro* by analyzing the expression of DC maturation markers CD80, CD86, CD40, and HLADR. To analyze the expression of DC maturation markers CD80, CD86, CD40, and HLADR, immature DCs (iDCs) were co-cultured with scFv-Erbitux-SNAP-IR700-irradiated OVCAR4 cells for 48 h. Flow cytometry analysis revealed that the scFv-Erbitux-SNAP-IR700-irradiated OVCAR4 cells slightly increased the expression of CD80 and CD86 on the surface of DCs, whereas the expression of CD40 and HLADR was significantly upregulated compared to scFv-Erbitux-SNAP-irradiated OVCAR4 cells (Figures 8A, 8B, S11, and S12) These data demonstrate that our NIR-PIT agent targeting EGFR can mature the iDCs that might trigger anticancer immunity. Similarly, the DC maturation was examined after co-culturing iDCs with scFv-Tisotumab-SNAP-IR700-irradiated SKOV3 cells for 48 h. The CD80 and CD86 expressions were significantly enhanced on the DCs co-cultured with scFv-Tisotumab-SNAP-IR700-treated SKOV3 cells compared to the control. In addition, the expressions of CD40 and HLADR were significantly increased on the surface of DCs stimulated by scFv-Tisotumab-SNAP-IR700-treated SKOV3 cells or lipopolysaccharide (LPS) compared to DCs co-cultured with scFv-Tisotumab-SNAP-treated SKOV3 (Figure 8C). The abovementioned results showed that our NIR-PIT agents could induce the maturation of iDCs, establishing the ability to augment the immune response.

## Discussion

Generally, ovarian cancer patients are diagnosed at a later stage. Surgery and platinum-based chemotherapy are the standard treatments for ovarian cancer patients. However, a high rate of recurrence has been found following the initial treatment. Therefore, NIR-PIT could be an alternative treatment strategy that uses nontoxic theranostic agents, which can be activated locally and on-demand using harmless light to kill the cancer cells and stimulate the host immune system against dead cancer cell antigens.^9^^,^^10^^,^^11^^,^^49^ In particular, NIR-PIT agents are generated through direct conjugation of mAb with IR700.^11^^,^^50^^,^^51^^,^^52^^,^^53^^,^^54^ The scFv contains the same antigen-binding domains of a whole antibody, and it can easily penetrate tumor masses because of its smaller size and more effective clearance from the circulation. It has a high specificity and affinity for antigens and low immunogenicity.^55^^,^^56^ In this study, we used scFv-SNAP-tag, targeting ovarian cancer cells expressing EGFR, Her2, FOLR1, TROP2, and TF antigen to generate 1:1 antibody IR700 ratio, which is important to get robust pharmacokinetic, efficacy, and safety profiles.^40^^,^^41^^,^^47^ Similar to other reports, five scFv-SNAP-tag proteins were successfully generated that demonstrated the rapid conjugation with different BG derivatives (SNAP-Surface Alexa Fluor 647 or BG-IR700) within 2 h.^41^^,^^42^ A key advantage of our approach is the use of SNAP-tag-mediated conjugation, which allows site-specific and uniform labeling, unlike conventional random chemical conjugation of IR700 to antibodies. This uniformity improves reproducibility and may enhance therapeutic performance.^57^^,^^58^^,^^59^ Moreover, the reduced molecular size of scFv-SNAP conjugates instead of using mAb-IR700 supports improved tumor penetration and lower background toxicity.^60^

In NIR-PIT, specific binding of NIR-PIT agents to the targeted tumor site is a pivotal step in achieving efficient therapeutic efficacy and limiting off-target effects.^11^ We initially confirmed strong and specific binding of scFv-SNAP-tag proteins to ovarian cancer cells, which is in line with other reports.^40^^,^^61^ In addition, the TF targeting scFv-Tisotumab-SNAP-IR700 showed specific membrane and internalized IR700 florescence signals in SKOV3, OVCAR3, and OVCAR4. The reason could be that scFv-Tisotumab-SNAP-IR700 was rapidly internalized into lysosomes. However, several studies demonstrated that internalization of NIR-PIT agents in cells was not necessary for the cell killing of targeted tumors.^62^^,^^63^ The membrane binding of NIR-PIT agents is the most important factor for their biological efficacy.^11^ Moreover, the expression patterns of EGFR, Her2, FOLR1, TROP2, and TF in 25 samples of human ovarian cancer tissues was simultaneously visualized using mIF assay. The NIR-PIT agents, developed for targeted therapy, were shown to specifically bind to ovarian cancer tissues, even with slight alterations to the commercially available antibodies, which was also observed in breast cancer studies.^57^^,^^59^ This suggests robust targeting capability and strongly supports the potential of NIR-PIT as a targeted therapy in ovarian cancer. However, a limitation of our study is the lack of systematic evaluation of antigen expression in normal tissues, which is important for predicting potential off-target effects. To address this, future work needs to be conducted including calculating tumor specificity scores by combining direct experimental validation with publicly available datasets, providing a clearer understanding of tumor-normal expression differences and helping to guide *in vivo* toxicity studies and clinical translation of the NIR-PIT agents.

The efficacy of NIR-PIT agents differs in studies due to the different light doses, NIR-PIT agents, cell lines, conjugation methods, and incubation time, translating to a large range of IC_50_ from nanomolar to micromolar.^35^^,^^40^^,^^41^^,^^64^^,^^65^^,^^66^ After light irradiation, the IR700-conjugated scFv-SNAP-tag proteins showed specific cytotoxicity in a concentration-dependent manner in high-expressing cell lines,^35^^,^^40^^,^^41^ which is in line when NIR-PIT was applied in different types of cancers *in vitro* and *in vivo*.^61^^,^^66^^,^^67^^,^^68^^,^^69^ The IC_50_ was 42 nM–1,420 nm at 2 J/cm^2^ light dose as several studies reported that high light doses (50 J/cm^2^) cause edema.^62^^,^^65^^,^^70^ Therefore, a relatively low NIR light dose combined with a higher concentration of NIR-PIT agents (1,600 nM) was selected based on our preliminary optimization experiments and previously published studies using IR700-conjugated agents in similar photoimmunotherapy settings, with the goal of balancing therapeutic efficacy and minimizing side effects.^14^^,^^71^^,^^72^ Further *in vivo* studies are required to refine the optimal irradiation conditions for clinical translation. The clinical relevance of IC_50_ values within the low nanomolar range may be further improved by using higher-affinity antibody fragments, enhancing tumor penetration, or refining dosing regimens to ensure clinically achievable concentrations.

One limitation of NIR-PIT is the restricted penetration depth of near-infrared light, which may reduce efficacy against deep-seated or bulky ovarian tumors.^13^^,^^73^ Potential strategies to overcome this include fiber optic probes, cylindrical diffusers, and laparoscopic or endoscopic approaches for intraperitoneal illumination.^68^^,^^74^^,^^75^^,^^76^ Repeated treatments may be necessary to achieve durable tumor control, especially in heterogeneous or bulky tumors, and dosing should be optimized to balance sufficient tumor accumulation with efficient light delivery, while minimizing systemic exposure.^77^^,^^78^^,^^79^ Future studies will focus on defining optimal dosing intervals, irradiation schedules, and combination strategies to maximize efficacy while maintaining safety.

We also confirmed the photocytotoxicity mediated by NIR-PIT agents using annexin assay. Consistent with previous studies, the flow cytometric dot plots exhibited that a significant percentage (46–68%) of cell death occurred by secondary necrosis/late apoptotic/necroptosis with 24%–39% of early apoptotic cell death in high-expressed cell lines.^14^^,^^34^^,^^61^^,^^66^^,^^80^^,^^81^ Our results suggest that all NIR-PIT agents cause targeted cell death in which most representing cells are in the necroptosis stage of cell death.

NIR-PIT causes rapid target-specific cell death through photochemical changes of antibody IR700-bound cells, thereby damaging the cell membrane of targeted cell. The pathway of cell death is not clear, although many studies demonstrated that necrotic cell death occurred after NIR-PIT.^14^^,^^18^^,^^34^^,^^65^ Previous studies demonstrated that necrosis could take place in a well-regulated and genetically guided way. Regulated necrosis has different forms including necroptosis, ferroptosis, pyroptosis, and autophagic cell death.^82^ EGFR- and Her2-targeting scFv-SNAP-based NIR-PIT induced cell death was suppressed by 18%–26% and 26%–27% when OVCAR4 and OVCAR3 cells were pretreated with necroptosis and ferroptosis inhibitors. However, apoptotic inhibitors rescued only 6%–15% cell death (Figure 6). This indicates that most cell death (53%) occur in regulated necrosis (necroptosis and ferroptosis) and other major cell death might be irreversible necrosis^77^^,^^83^ that could not be rescued by these inhibitors. In addition, 5% cell death was rescued when IGROV1 and Hey cells were pretreated with necroptosis and ferroptosis inhibitors. The reason might be that scFv-Farletuzumab-SNAP-IR700 and scFv-Sacituzumab-SNAP-IR700 were highly cytotoxic to IGROV1 and Hey, respectively, as we described in Results section (Figure 4). Similar to mAb-IR700, our scFv-SNAP-based NIR-PIT agents could also localize in the lysosome and may induce lysosomal membrane damage and leakage of the lysosomal contents into the cytosol.^14^ Lysosomal damage or other cell death modalities such as pyroptosis have not been analyzed in this study, and further investigation is needed. Membrane-bound or internalized scFv-SNAP-based NIR-PIT agents might induce massive damage to membranes or lysosomes, leading to irreversible necrosis that could not be rescued by the cell death inhibitors. These results indicate that multiple coexistent cell death mechanisms may occur in scFv-SNAP-based NIR-PIT, which is in line with other previous result when affibody-based NIR-PIT was used.^61^ The relative contribution of apoptosis, necroptosis, ferroptosis, or other mechanisms may depend on both the targeted molecule and the specific cell line. The types of cell and target have influence on the subcellular localization of NIR-PIT agents. This might be the reason why universal results have not been obtained concerning the involvement of cell death rescuing by cell death inhibitor in NIR-PIT.

NIR-PIT selectively kills target cells and induces ICD.^18^ Kobayashi’s group previously demonstrated cell surface expression of CRT, HSP70, and HSP90 and a rapid release of ATP and HMGB1, when tumors are treated with Her2-targeting NIR-PIT.^18^^,^^77^ In this study, we demonstrated the major features of ICD after NIR-PIT including cell surface exposure of CRT, HSP70, and HSP90 and extracellular release of ATP and HMGB1. We observed 1.4- to 3.2-fold, 2- to 8.5-fold, and 5.4- to 119-fold enhanced expression of calreticulin, HSP70, and HSP90, respectively in high-expressing cells treated with scFv-SNAP-IR700 compared to the control, which was consistent with other reports.^34^^,^^61^^,^^80^^,^^84^ These results indicate that all five NIR-PIT-treated cells augmented the expression of cell surface calreticulin, HSP70, and HSP90. The expression of HSP90 was higher compared to HSP70 expression on all five NIR-PIT-induced dying cells, which was in line with previously published studies.^18^^,^^63^ The extracellular levels of ATP and HMGB1 in the culture supernatants of treated ovarian cancer cells were evaluated in this study. Consistent with other reports, we observed high level of extracellular ATP and HMGB1 releases after all five scFv-based NIR-PITs.^18^^,^^34^^,^^61^ In contrast, Hey cells (treated with scFv-Sacituzumab-SNAP-IR700) released 74.2 pmol ATP and 13.8 ng/mL HMGB1, which was the lowest among all five treatments. The probable reason might be the morphological difference of different cell lines. A recent study reported that the serum concentration of HMGB1 increased after NIR-PIT in head and neck squamous cell carcinoma patients.^85^ This study also provides proof of principle that scFv-SNAP-tag-based NIR-PIT agents can induce ICD and that further *in vivo* studies need to be conducted.

The effectiveness of the cancer treatment strategy can be enhanced if the treatment can remove the cancer cell specifically and induce host cell immunity. NIR-PIT can directly kill cancer cells, and the cancer-associated antigen released from cancer cells further processes and matures the DCs.^18^^,^^50^^,^^86^ As our NIR-PIT agents induce ICD by exposing DAMPs, we expected that these DAMPs could activate and mature DCs, priming anti-tumor immunity.^18^^,^^61^ We demonstrated that the expressions of CD80, CD86, CD40, and HLADR were 2.4-, 3.6-, 7.7-, and 10.4-fold, respectively, higher in DCs co-cultured with scFv-Tisotumab-SNAP-IR700-treated SKOV3 cells compared to the scFv-Tisotumab-SNAP-treated SKOV3 cells. This was in line with previously published studies demonstrating that Her2-targeting affibody increased the expression of CD80, CD86, HLADR, and CD40.^18^^,^^61^ However, the expressions of CD80 (1.9-fold), CD86 (1.7-fold), and HLADR (4.9-fold) were slightly lower in co-cultured DCs with scFv-Erbitux-SNAP-IR700-treated OVCAR4 cells than co-cultured DCs with scFv-Tisotumab-SNAP-IR700-treated SKOV3 cells. Moreover, we observed 12.1-fold higher MFI of CD40 compared to the control. These findings indicate that our NIR-PIT agents can effectively induce DC maturation, suggesting the potential to generate long-lasting anti-tumor immunity in ovarian cancer patients. Although downstream functional assays of adaptive immune activation (e.g., T cell proliferation and cytokine release) were not performed in this study, future work will include such assays and extend these findings to *in vivo* models to evaluate systemic immune activation in the context of a complete immune system. Importantly, the ability of NIR-PIT to induce ICD provides a strong rationale for combination with immune checkpoint inhibitors such as anti-PD-1/PD-L1 or anti-CTLA-4 antibodies, which may enhance and prolong anti-tumor responses in ovarian cancer.^19^^,^^86^^,^^87^

Another limitation of this study is that the evaluation of NIR-PIT agents was restricted to *in vitro* cell culture models and *ex vivo* human ovarian cancer tissue samples. Aspects such as absorption, distribution, metabolism, and excretion of the agents, as well as the influence of immune-suppressive cells, extracellular matrix components, limited light penetration, and potential tissue toxicity *in vivo*, cannot be fully recapitulated under *in vitro* conditions. To address this, future studies employing ovarian cancer xenograft or orthotopic models will be essential to evaluate biodistribution, therapeutic efficacy, and safety, thereby advancing the clinical translation of these agents. In addition, while inhibitor experiments indicated the involvement of apoptosis, necroptosis, and ferroptosis in NIR-PIT-induced cell death, protein-level validation of key markers such as cleaved caspase-3, cleaved PARP, phosphorylated RIPK3, and phosphorylated MLKL was not performed. Despite this limitation, our findings provide mechanistic insight into the pathways of regulated cell death triggered by NIR-PIT and establish a foundation for future molecular analyses to further confirm these mechanisms.

In conclusion, this study provides proof-of-concept evidence that SNAP-tag-based NIR-PIT agents specifically target ovarian cancer antigens, induce ICD, and promote DC maturation, thereby offering potential for both direct tumor cytotoxicity and activation of anti-tumor immunity. These findings lay the groundwork for future preclinical *in vivo* studies to refine therapeutic efficacy and safety, while also highlighting the advantages of SNAP-tag technology for generating highly defined conjugates. Ultimately, this approach may support pre-screening of patients, enable real-time monitoring of treatment responses, and facilitate personalized application of NIR-PIT in ovarian cancer.

## Material and methods

### Cell culture

Ovarian cancer cell lines SKOV3 (HTB-77), OVCAR3 (HTB-161), IGROV1 (SCC-203), and A2780 (ECACC-93112519) and human embryonic kidney cell line HEK293T (CRL-11268) were purchased from American Type Culture Collection, European Collection of Authenticated Cell Cultures, and Sigma-Aldrich. OVCAR4 and Hey cell lines were kindly provided by Dr. Karen Bräutigam (Department of Gynecology and Obstetrics, University Hospital Schleswig-Holstein, Campus Lübeck) as a gift. All cell lines were authenticated and tested negative for mycoplasma contamination (Eurofins Genomics, Germany) prior to use. These cell lines were cultured in RPMI 1640 (Biowest) culture medium supplemented with 10% (v/v) fetal bovine serum (FBS) (Thermo Fisher Scientific) and 1% (v/v) penicillin and streptomycin (Thermo Fisher Scientific). All cells were cultured in an incubator at 37°C in a humidified atmosphere containing 5% CO_2_ for no more than 30 passages.

### Expression and enrichment of scFv-SNAP fusion proteins

The scFv-SNAP-containing HEK293T cells were cultured in RPMI 1640 complete medium supplemented with 0.1% (v/v) zeocin (InvivoGen) to keep the transfected cells selection. Culture supernatant was collected and used for protein enrichment. Äkta FPLC system (GE Healthcare Bio-Sciences AB, Uppsala, Sweden) and Ni-NTA Superflow (Qiagen, Hilden, Germany) cartridge were used to purify the C-terminal 6× His-tagged fusion proteins from cell-free supernatants. The proteins were enriched by following the protocol of Hussain et al. 2019.^47^ All the eluted fractions were incubated with SNAP-Surface Alexa Fluor 488 for 20 min in the dark at room temperature and loaded in 10% SDS gel along with blue pre-stained protein standard broad range (New England Biolabs) at 160 V for 60 min. After separation, labeled proteins were visualized either with a UV transilluminator Gel Doc XR gel documentation system or Odyssey DLx Imager to confirm the activity of SNAP-tag by visualizing Alexa Fluor 488 signals and the presence of proteins followed by Coomassie brilliant blue staining. The protein concentration was determined by BSA standards (New England Biolabs, 20 mg/mL) using Image Lab software.

### Modification of IR700 with BG

IR700 (LI-COR Biosciences, Lincoln, NE, USA. #92970010) and BG-PEG-NHS (New England Biolabs, Ipswich, MA, USA, #S9150S) were dissolved to 1.0 nM and 10 nM in DMSO respectively, followed by incubating at a 1:2 molar ratio at room temperature for 2 h. Conjugated IR700 and BG-PEG-NHS was analyzed and purified by HPLC according to Hossain et al*.*, 2019.^47^ The mass of BG-IR700 was confirmed using a Bruker micrOTOF LC mass spectrometer with an electrospray ion source. All HPLC and mass spectrometry analyses were done by the HPLC facility, Institute of Organic Chemistry, Justus-Liebig-University Giessen.

### Conjugation of SNAP-tag fusion proteins with SNAP-Surface Alexa Fluor 488 or BG-IR700

SNAP-Surface Alexa Fluor 488 or BG-IR700 was conjugated to SNAP-tag fusion proteins by incubating at a 2:1 molar ratio at room temperature in dark for 2 h. The residual dyes were removed by 40K MWCO Zeba Spin Desalting Columns according to manufacturer’s protocol. The labeled proteins were visualized after separation by SDS-PAGE and the concentration was measured as described in Results section.

### Expression-level analysis of cell surface antigens in ovarian cancer cells

Cells (4 × 10^5^) were treated with anti-EGFR (EGFR mAb, clone H11, 0.5 μg, Invitrogen, #MA5-13269), anti FOLR1 (FOLR1 mAb, clone 548908, 0.5 μg, Invitrogen, #MA5-23917), anti-TROP2 (TROP2 mAb, clone MR54, 1 μg, Invitrogen, #14-6024-82), and anti TF (CD142 mAb, clone HTF-1, 10 μL, Miltenyi Biotec, #130-098-741) antibodies for 30 min on ice. As the anti-HER2 antibody (ErbB2 mAb, clone 3B5) targets the intracellular domain, HER2 staining was performed after permeabilization by following the manufacturer instruction. Cells were fixed by 4% formaldehyde followed by permeabilization with 0.1% Triton X-100 in TBS for 5 min. Then, blocking buffer (10% FBS and 1% BSA in PBS) was added and cells were incubated on ice for 30 min. Cells were treated with anti-Her2 (ErbB2 mAb, 3B5, Invitrogen, #MA5-13675) antibody. After washing twice, cells were incubated with goat anti-mouse IgG (H + L) highly cross-adsorbed secondary antibody conjugated with Alexa Fluor Plus 647 (0.25 μg, Invitrogen, #A32728) for 30 min on ice. After washing twice, cells were resuspended and analyzed by CytoFLEX S Flow Cytometer. Data were analyzed in FlowJo 10.7.1 from three independent experiments. The ΔMFI was calculated from the MFI of the cells expressing the marker of interest divided by the MFI of the unstained cells.

### Analysis of binding specificity of NIR-PIT agents by flow cytometry

SKOV3, OVCAR3, IGROV1, A2780, OVCAR4, and Hey were used to analyze the binding efficiency of 647-conjugated scFv-Erbitux-SNAP, scFv-Hereceptin-SNAP, scFv-Farletuzumab-SNAP, scFv-Tisotuzumab-SNAP, and scFv-Sacituzumab-SNAP. Cells were washed and stained with 1 μg scFv-SNAP-647 with PBS for 30 min on ice. Cells were washed twice and resuspended and analyzed by CytoFLEX S Flow Cytometer. In addition, IR700-conjugated scFv-Hereceptin-SNAP, scFv-Farletuzumab-SNAP, scFv-Tisotuzumab-SNAP, and scFv-Sacitumab-SNAP were used to confirm the specific binding using same flow cytometry setup and analyzed by CytoFLEX LX Flow Cytometer.

### Binding property analysis by fluorescence microscopy

Fluorescence microscopy was used to confirm the binding of IR700-conjugated fusion proteins to ovarian cancer cell lines. Cells were seeded in black 96-well plate with a clear bottom to a density of 40,000 cells/well and incubated overnight at 37°C. Cells were washed with PBS twice and then incubated with 1 μg of each scFv-SNAP-IR700 on ice for 30 min. Then cells were washed with PBS twice, followed by incubating with Hoechst 33342 fluorescent nuclear counterstain (1:500 in PBS) (Thermo Fisher Scientific) for 10 min at room temperature. Cells were washed with PBS and then visualized with a DMi8 S Live-cell microscope using a 100× oil objective.

### Multiplex immunofluorescence

Ovarian cancer tissue microarray was heated at 60°C for 40 min and subsequently placed in xylene for 20 min. The sections were then rehydrated in descending concentrations of ethanol starting from 100% (2 × 5 min), 90% (5 min), 80% (5 min), and 70% (5 min) and last washed twice with deionized water. Next, the sections were placed in slide holders containing retrieval buffer (sodium citrate buffer, pH 6.0) and boiled in a microwave oven for 20 min. Then, the sections were placed in 3% hydrogen peroxide for 10 min and subsequently washed three times with water followed by blocking in blocking buffer (10% goat serum in PBS) for 1 h. Next, the sections were drop incubated with primary antibody (FOLR1 antibody, clone 548908, 1:50, Invitrogen, #MA5-23917) overnight at 4°C in a humidified chamber. The following day, the sections were placed at room temperature for 1 h and washed three times in PBST (PBS with 0.1% TWEEN 20) and subsequently incubated with secondary antibody conjugated with horseradish peroxidase (HRP) (anti-mouse IgG HRP, Invitrogen, #G21040) for 1 h. The sections were washed three times in PBST and followed by incubation with iFluor 430 Tyramide (AAT Bioquest, #45096) for 10 min and subsequently washed three times in PBST. The sections were then placed in slide holders containing stripping buffer (sodium citrate buffer, pH 6.0) and boiled in a microwave oven for 20 min. The slides were stained with anti-Her2 antibody (ErbB2 mAb, 3B5, 1:500, Invitrogen, #MA5-13675), anti-TF antibody (CD142 mAb, clone HTF-1, 10 μL, Miltenyi Biotec, #130-098-741), anti-EGFR antibody (Sigma-Aldrich, 1:200, #HPA018530), and anti-TROP2 antibody (TROP2 mAb, clone MR54, 1:100, Invitrogen, #14-6024-82), followed by incubation with secondary antibody (anti-mouse or anti-rabbit according to primary antibody). After that the sections were incubated with Alexa Fluor 488 Tyramide (Invitrogen, B40953), Alexa Fluor 546 Tyramine (Invitrogen, B40954), Alexa Fluor 647 Tyramide (Invitrogen, B40958), iFluor 750 Styramide (AAT Bioquest, #45065) according to the abovementioned procedure. Finally, the slides were prepared for fluorescence imaging by mounting them with mounting media with DAPI (#H-1200, Vector Laboratories). For binding analysis of scFv-SNAP fusion proteins, scFv-Erbitux-SNAP, scFv-Hereceptin-SNAP, scFv-Farletuzumab-SNAP, scFv-Tisotuzumab-SNAP, and scFv-Sacituzumab-SNAP were conjugated with SNAP-biotin (New England Biolabs, #S9110S) by incubating at a 1:3 molar ratio at room temperature for 2 h. Tissue staining was performed according to the above-mentioned procedure except instead of using secondary antibody, streptavidin-conjugated HRP was used. Axioscan 7 (Zeiss, ZEN blue software) was used for fluorescence imaging. Data were analyzed using the open-source digital pathology software QuPath.^88^

### Photocytotoxicity of NIR-PIT agents

SKOV3, OVCAR3, IGROV1, A2780, OVCAR4 and Hey cells were seeded in 96-well plates at a density of 5,000 cells/well and incubated at 37°C overnight. The cells were washed with serum-free medium twice, followed by incubation with serially diluted IR700-conjugated and -unconjugated scFv-SNAP fusion proteins at 12.5, 25, 50, 100, 200, 400, 800, and 1,600 nM at 37°C for 4 h in the dark. Cells incubated with media or zeocin were set as negative or positive control. A red LED, which emits light at 670–710 nm wavelength (L690-66-60; Marubeni America Co., New York, NY) was used to irradiate the cells with LED power density 20 μW/cm^2^ at 400 mA CW (measured with an optical power meter, PM100; Thorlabs, Newton, NJ). After NIR light irradiation (2 J/cm^2^), the cells were cultured in complete medium for 24 h at 37°C and 5% CO_2_ in the dark. Cell viability was determined by incubating cells with a 50 μL XTT (Cell Proliferation Kit II, Roche #11465015001) labeling mixture at 37°C for 4 h. Reduction of XTT to formazan by viable tumor cells was monitored at a 450 nm absorbance wavelength and 650 nm reference wavelength using an Infinite Mplex microplate reader. The data were analyzed in GraphPad software using dose-response curve.

### Induction of cell death by NIR-PIT agents

After the treatment with NIR PIT agents, cell death induction was determined using SNAP-Surface Alexa Fluor 647-conjugated annexin V-SNAP fusion protein and propidium iodide according to Woitok et al*.* 2020.^48^ SKOV3, OVCAR3, IGROV1, A2780, OVCAR4, and Hey cells were seeded in 24-well plates at a density of 50,000 cells/well in triplicates and incubated at 37°C overnight. The cells were washed with serum-free medium twice, followed by incubation with serially diluted IR700-conjugated and -unconjugated scFv-SNAP fusion proteins at 1,600 nM at 37°C for 4 h in the dark. Cells incubated with media or zeocin were set as negative or positive control. After 24-h NIR light irradiation (2 J/cm^2^), cells were harvested and treated with 0.5 μg of annexin V-SNAP-647 in annexin-binding buffer at room temperature for 30 min. Cells were washed with annexin-binding buffer once and resuspended and followed by incubating with 0.1 μg of propidium iodide (Thermo Fisher Scientific) at room temperature for 10 min. The necrotic, early apoptotic, and late apoptotic/necroptotic cells were detected on CytoFLEX S Flow Cytometer. The gating strategy is described in Figure S13A.

### Determining the type of cell death induced by NIR-PIT agents

To determine the type of cell death induced by NIR-PIT reagents in ovarian cancer cells, we used three different cell death inhibitors that specifically block apoptosis (zVAD-fmk, 25 μM, Invivogen, #tlrl-vad), necroptosis (Necrostatin-1, 20 μM, AdipoGen life Sciences, #AG-CR1-2900-M005), and ferroptosis (Ferrostatin-1, 1 μM, Cayman Chemical Company, #17729). In this experiment, we used one cell line for each NIR-PIT agent (OVCAR4 cells for scFv-Erbitux-SNAP-IR700, OVCAR3 for scFv-Herceptin-SNAP-IR700, IGROV1 for scFv-Farletuzumab-SNAP-IR700, Hey for scFv-Sacituzumab-SNAP-IR700, and SKOV3 for scFv-Tisotumab-SNAP-IR700). Cells were incubated with 1,600 nM NIR-PIT agent for 4 h at 37°C in the dark. After 3 washing steps with PBS, fresh phenol red-free culture medium containing zVAD-fmk (25 μM, Invivogen, #tlrl-vad) or Necrostatin-1 (20 μM, AdipoGen Life Sciences, #AG-CR1-2900-M005), or Ferrostatin-1 (1 μM, Cayman Chemical Company, #17729) were added to the cells and incubated for 30 min. The cells were treated with NIR light as described in Results section. XTT assay was performed to determine the cell-inhibiting ability of the cell death inhibitor. The percentage of cell death inhibition was calculated by subtracting the percentage of cell viability with the respective inhibitor from the percentage of cell viability without the inhibitor.

### Calreticulin, HSP70, and HSP90 assay by flow cytometry

SKOV3, OVCAR3, IGROV1, A2780, OVCAR4, and Hey cells were seeded in 24-well plates at a density of 50,000 cells/well in triplicates and incubated at 37°C overnight. Cells were incubated with IR700-conjugated and -unconjugated scFv-SNAP fusion proteins in 1,600 nM at 37°C for 4 h in the dark. Cells exposed to NIR light irradiation without incubation with NIR-PIT agents were used as control. After 24-h NIR light irradiation, cells were harvested and incubated with 0.50 μg calreticulin (488-conjugated, Clone 681233, R&D Systems #IC38981G-100UG) antibody or 2 μL HSP70 (FITC-Conjugated, Miltenyi Biotech #130-105-548) antibody, or 1 μg HSP90 (APC conjugated, H9010, Invitrogen #MA5-45102) antibody for 30 min at 4°C. After washing twice, cells were resuspended and analyzed by CytoFLEX S Flow Cytometer. The number of the cells was expressed in percentages from the living gated cells, and the HSP70-specific MFI was calculated. The MFI values are divided by 1,000 and shown in bar graph and used for statistical analysis. The data were analyzed in FlowJo and GraphPad software. The gating strategy is described in Figure S13B.

### ATP and HMGB1 assay

For analyzing extracellular release of ATP and HMGB1 level after the treatment, the same experimental setup was used as described in Results section. After 24 h, the culture supernatants were collected and centrifuged. Extracellular ATP concentrations in the culture supernatants were evaluated by a luciferin-based ATP assay kit (ENLITEN, Promega, Madison, WI, USA, # 0000410249) according to the manufacturer’s instructions. The concentration of HMGB1 in the supernatant was measured using an HMGB1 ELISA kit (IBL International, Hamburg, Germany, # EHMG153) according to the manufacturer’s instructions. The data were analyzed in GraphPad software.

### Measurement of CD80, CD86, CD40, and HLADR expression level by flow cytometry

Peripheral blood mononuclear cells (PBMCs) were purchased from BPS Bioscience (#79059). CD14^+^ monocytes cells were isolated from PBMCs by positive selection using CD14 microbeads (Miltenyi Biotec #130-097-052) and MACS column (LS column, Miltenyi Biotec #130-042-401) as described by Ogawa et al., 2017. The efficiency of CD14^+^ monocytes cells isolation was investigated by staining with CD14 antibody (APC conjugated, clone REA599, Miltenyi Biotec #130-110-578). iDCs were generated by culturing the CD14^+^ monocytes cells in the presence of 50 ng/mL granulocyte-macrophage colony-stimulating factor (GM-CSF) (PeproTech #300-03) and 10 ng/mL interleukin-4 (IL-4) (Miltenyi Biotec #130-093-915). The generation of iDCs was investigated by staining with DC marker (CD209 monoclonal antibody, B515 conjugated, clone REA617, Miltenyi Biotec #130-132-023). SKOV3 cells were incubated with scFv-Tisotumab-SNAP and scFv-Tisotumab-SNAP-IR700 with NIR exposure and co-cultured for 24–48 h with iDCs (day 5) at a DC/tumor cell ratio of 1:2. In addition, OVCAR4 cells were incubated with scFv-Erbitux-SNAP and scFv-Erbitux-SNAP-IR700 with NIR exposure and co-cultured for 24–48 h with iDCs (day 5) at a DC/tumor cell ratio of 1:2. DCs stimulated with 100 ng/mL of LPS (Invitrogen #00-4976-93) for 12 h were used as a positive control for DC maturation. After 48 h, all floating cells were collected and washed with PBS twice. Cells were incubated with 1.0 μg of CD80 (APC conjugated, clone B7-1, Invitrogen #17-0809-42) antibody and 2 μL of CD86 (PE conjugated, clone FM9-5, Miltenyi Biotec #130-094-877) antibody, 1.0 μg of CD40 (FITC conjugated, clone MH40-3, Invitrogen #11-0402-82) antibody, and 2 μL of HLADR (APC conjugated, clone REA805, Miltenyi Biotec #130-111-943) antibody for 30 min on ice. After washing twice, cells were resuspended and analyzed by CytoFLEX S Flow Cytometer. The number of the cells was expressed in percentages from the living DC-gated cells (Figure S13C), and the CD80-, CD86-, CD40-, and HLADR-specific MFI was calculated for statistical analysis.

### Statistics

All results were reported as means with SD. For multiple comparisons, one-way ANOVA and Welch ANOVA with a Dunnett’s multiple comparisons test were used. For two-treatment analysis, an unpaired *t* test was used. *p* values of less than 0.05 were considered significant. The analyses were performed with GraphPad software version 9.0.0 (GraphPad Software Inc., La Jolla, CA, USA).

## Data availability

The data associated with this manuscript are included in the manuscript and in the supplemental information. Any additional information is available upon reasonable request.

## Acknowledgments

This work was funded in part by a Research Grant of the University Medical Center Giessen and Marburg (UKGM) (8/2021 GI). We acknowledge the HPLC facility in Justus-Liebig-University Giessen for performing all HPLC and mass spectrometry analysis.

## Author contributions

T.M.M., investigation, writing – original draft preparation; C.Z., investigation; W.S., investigation; M.A.-R., investigation and resources; R.S., M.N., N.E.-M., and F.Z., visualization and writing – review & editing; I.M.-H., conceptualization, supervision, writing – review & editing; A.F.H., conceptualization, funding acquisition, investigation, supervision, and writing – review & editing.

## Declaration of interests

T.M.M. reports financial support was provided by Research Grant of the University Medical Center Giessen and Marburg (UKGM).

## Supplemental information

Document S1. Figures S1–S13 and Tables S1–S4

Document S2. Article plus supplemental information
