# Supplementary material for: Near-infrared photoimmunotherapy for effective elimination of ovarian cancer cells by inducing immunogenic cell death
Source: Mol Ther Oncol. 2025 Nov 12;33(4):201086. doi: 10.1016/j.omton.2025.201086 (PMC12681927; doi:10.1016/j.omton.2025.201086)
Supplement: Document S1. Figures S1–S13 and Tables S1–S4 [file mmc1.pdf]

OMTON, Volume *33*

Supplemental information

Near-infrared photoimmunotherapy for effective elimination of ovarian cancer cells by inducing immunogenic cell death

T.M. Mohiuddin, Chaoyu Zhang, Wenjie Sheng, Marwah Al-Rawe, Roland Schmitz, Marcus Niebert, Natalia El-Merhie, Felix Zeppernick, Ivo Meinhold-Heerlein, and Ahmad Fawzi Hussain

Supplemental Materials

Table S1: Plasmids used in section 2.1.

Plasmids	Genotype	References
pMS-scFv-425-SNAP	ori ColE1, Amp^R, $P_{CMV,}$ IgG leader, scFv_425, SNAP tag, IRES, EGFP, f1 ori, and $Bleo^R$	[49]
pMS-scFv-Erbitux-SNAP	ori ColE1, Amp^R, $P_{CMV,}$ IgG leader, scFv_Erbitux, SNAP tag, IRES, EGFP, f1 ori, and $Bleo^R$	this study
pMS-scFv-Herceptin-SNAP	ori ColE1, Amp^R, $P_{CMV,}$ IgG leader, scFv_Herceptin, SNAP tag, IRES, EGFP, f1 ori, and $Bleo^R$	this study
pMS-scFv-Farletuzumab-SNAP	ori ColE1, Amp^R, $P_{CMV,}$ IgG leader, scFv_Farletuzumab, SNAP tag, IRES, EGFP, f1 ori, and $Bleo^R$	this study
pMS-scFv-Sacituzumab-SNAP	ori ColE1, Amp^R, $P_{CMV,}$ IgG leader, scFv_Sacituzumab, SNAP tag, IRES, EGFP, f1 ori, and $Bleo^R$	this study
pMS-scFv-Tisotumab-SNAP	ori ColE1, Amp^R, $P_{CMV,}$ IgG leader, scFv_Tisotumab, SNAP tag, IRES, EGFP, f1 ori, and $Bleo^R$	this study

Table S2: Strains used in section 2.1.

Strain	Genotype	Reference
E. coli DH5α	-	[49]
E. coli DH5α pMS-scFv-Erbitux-SNAP	*E. coli* DH5α pMS-scFv-Erbitux-SNAP, Amp^R	this study
E. coli DH5α pMS-scFv-Herceptin-SNAP	*E. coli* DH5α pMS-scFv-Herceptin-SNAP, Amp^R	this study
E. coli DH5α pMS-scFv-Farletuzumab-SNAP	*E. coli* DH5α pMS-scFv-Farletuzumab-SNAP, Amp^R	this study
E. coli DH5α pMS-scFv-Sacituzumab-SNAP	*E. coli* DH5α pMS-scFv-Sacituzumab-SNAP, Amp^R	this study
E. coli DH5α pMS-scFv-Tisotumab-SNAP	*E. coli* DH5α pMS-scFv-Tisotumab-SNAP, Amp^R	this study

Table S3: HEK293T cells containing SNAP tag fusion protein plasmid used in section 2.1

Strain	Genotype	Reference
HEK293T	-	[49]
HEK293T-pMS-scFv-Erbitux-SNAP	HEK293T-pMS-scFv-Erbitux-SNAP, $Bleo^R$	this study
HEK293T-pMS-scFv-Herceptin-SNAP	HEK293T-pMS-scFv-Herceptin-SNAP, $Bleo^R$	this study
HEK293T-pMS-scFv-Farletuzumab-SNAP	HEK293T-pMS-scFv-Farletuzumab-SNAP, $Bleo^R$	this study
HEK293T-pMS-scFv-Sacituzumab-SNAP	HEK293T-pMS-scFv-Sacituzumab-SNAP, $Bleo^R$	this study
HEK293T-pMS-scFv-Tisotumab-SNAP	HEK293T-pMS-scFv-Tisotumab-SNAP, $Bleo^R$	this study

Table S4. Binding efficiency of five NIR-PIT agents in ovarian cancer cell lines

NIR-PIT agents	SKOV3	OVCAR3	IGROV1	A2780	OVCAR4	Hey
scFv-Erbitux-SNAP-IR700	high	high	medium	-	high	-
scFv-Herceptin-SNAP-IR700	high	high	-	-	-	-
scFv-Farletuzumab-SNAP-IR700	-	medium	high	-	-	-
scFv-Sacituzumab-SNAP-IR700	medium	high	-	-	high	high
scFv-Tisotumab-SNAP-IR700	medium	medium	-	-	medium	-

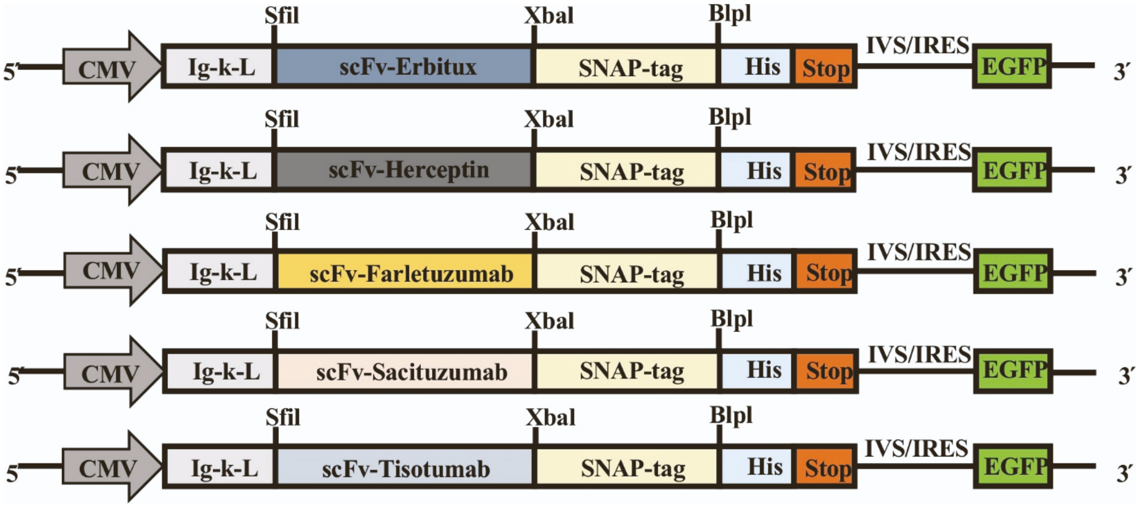

Figure S1: Construction and expression of the SNAP tag fusion proteins. (a) Schematic diagram of the scFv-SNAP tag constructs. CMV: cytomegalovirus promoter; IgK leader: immunoglobulin kappa chain leader; His: poly-histidine tag; Stop: TGA stop codon, IRES: internal ribosome entry site, EGFP: enhanced fluorescent protein.

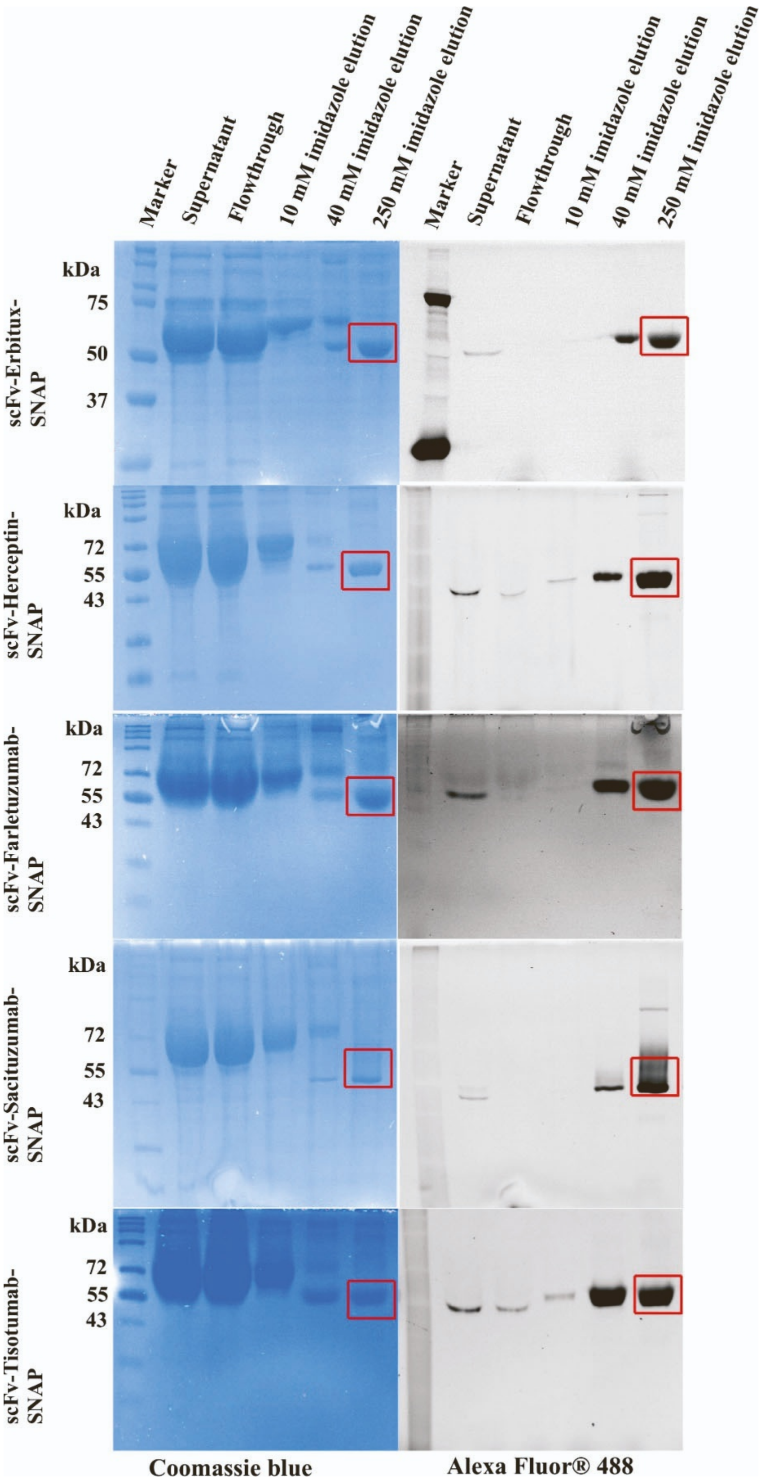

Figure S2: Enrichment of scFv-Herceptin-SNAP, scFv-Farletuzumab-SNAP, scFv-Sacituzumab-SNAP and scFv-Tisotumab-SNAP by nickel NTA using His-tagged and confirmed by SDS-PAGE by Coomassie blue staining (left panel) and SNAP-Surface® Alexa Fluor® 488 signal (right panel). The signal was visualized with ChemiDoc XRS^{+} System. Dual color protein standard broad range (10-250 kDa) or Protein standard broad range (11-250 kDa) were used as marker. The red box indicates the corresponding protein bands.

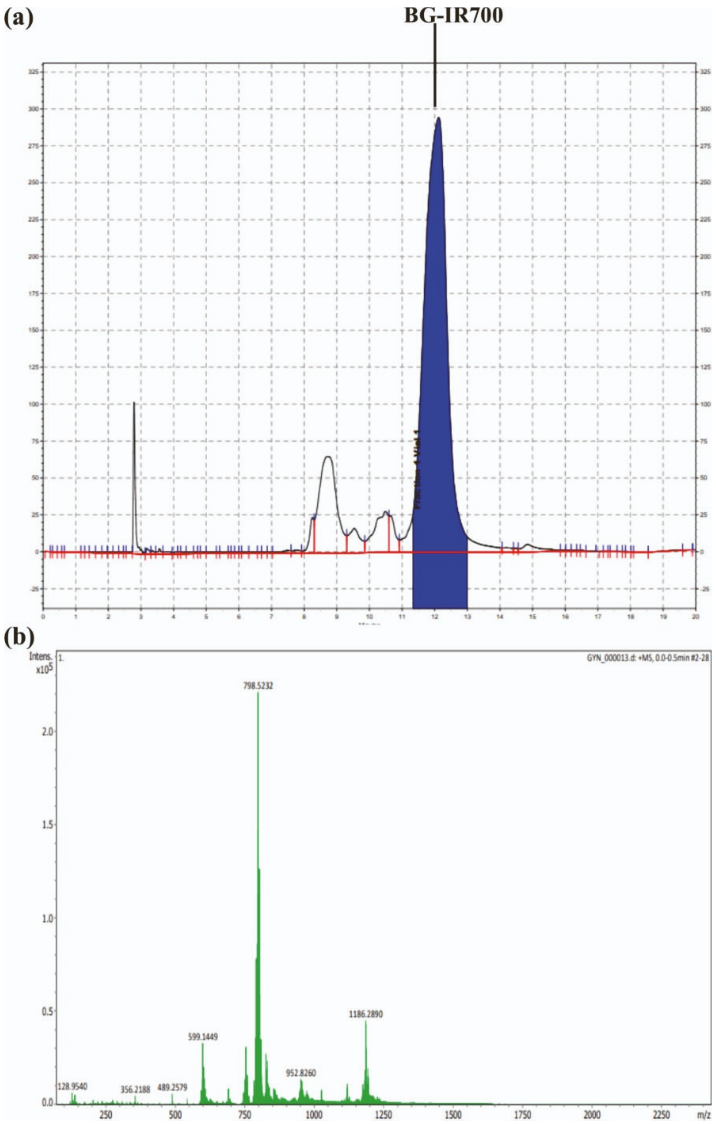

Figure S3: Purification of BG-IR700. (a) HPLC analysis of BG-IR700. The arrow indicates the peak of purified BG-IR700. (b) Mass spectra of BG-IR700.

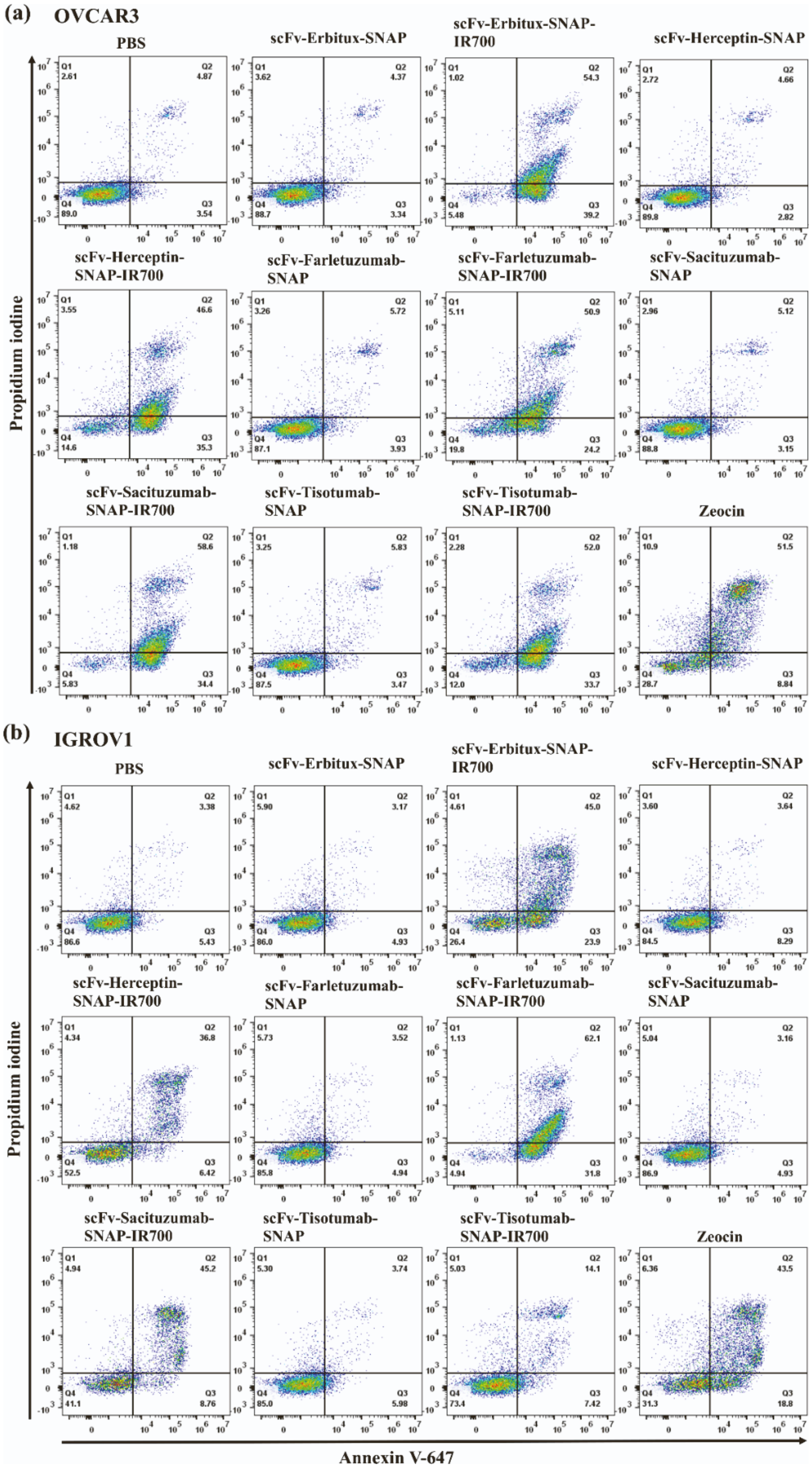

Figure S4: NIR-PIT agents mediated cell death in OVCAR3 and IGROV1 cells. Cell death was measured by annexin assay after 24 h treatment. The flow cytometric dot plots depicted the living (annexin V^{-}/PI^{-}, Q4), early apoptotic (annexin V^{+}/PI^{-}, Q3), late apoptotic/necroptotic (annexin V^{+}/PI^{+}, Q2) and necrotic (annexin V^{-}/PI^{+}, Q1) cells. Cells treated with unconjugated scFv-SNAP localized mostly to the bottom left quadrant (Q4, viable cells) and NIR-PIT agents treated cells localized mostly to the top right quadrant (Q2, Necroptotic/Late apoptotic cells).

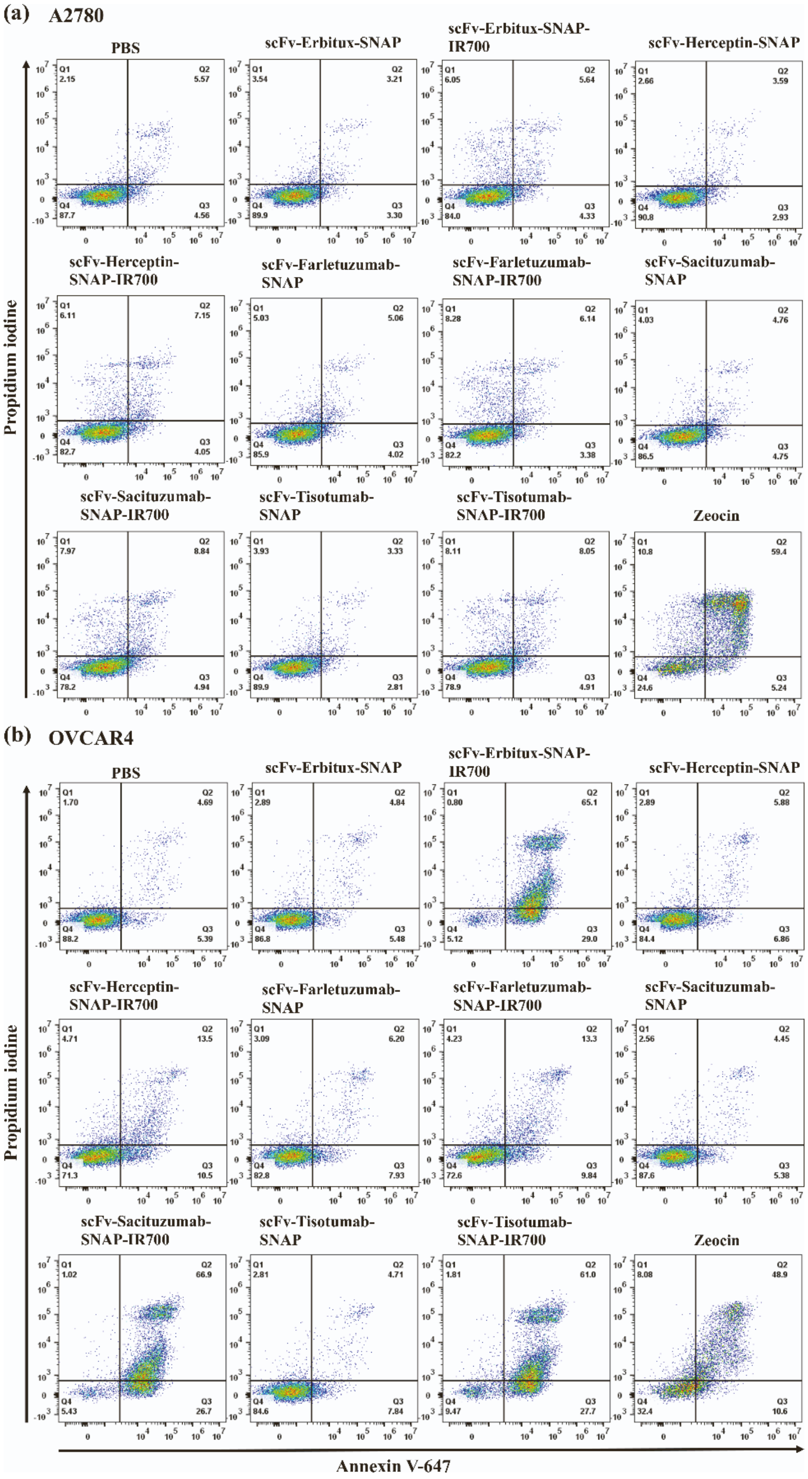

Figure S5: NIR-PIT agents mediated cell death in A2780 and OVCAR4 cells. Cell death was measured by annexin assay after 24 h treatment. The flow cytometric dot plots depicted the living (annexin V^{-}/PI^{-}, Q4), early apoptotic (annexin V^{+}/PI^{-}, Q3), late apoptotic/necroptotic (annexin V^{+}/PI^{+}, Q2) and necrotic (annexin V^{-}/PI^{+}, Q1) cells. Cells treated with unconjugated scFv-SNAP localized mostly to the bottom left quadrant (Q4, viable cells) and NIR-PIT agents treated cells localized mostly to the top right quadrant (Q2, Necroptotic/Late apoptotic cells).

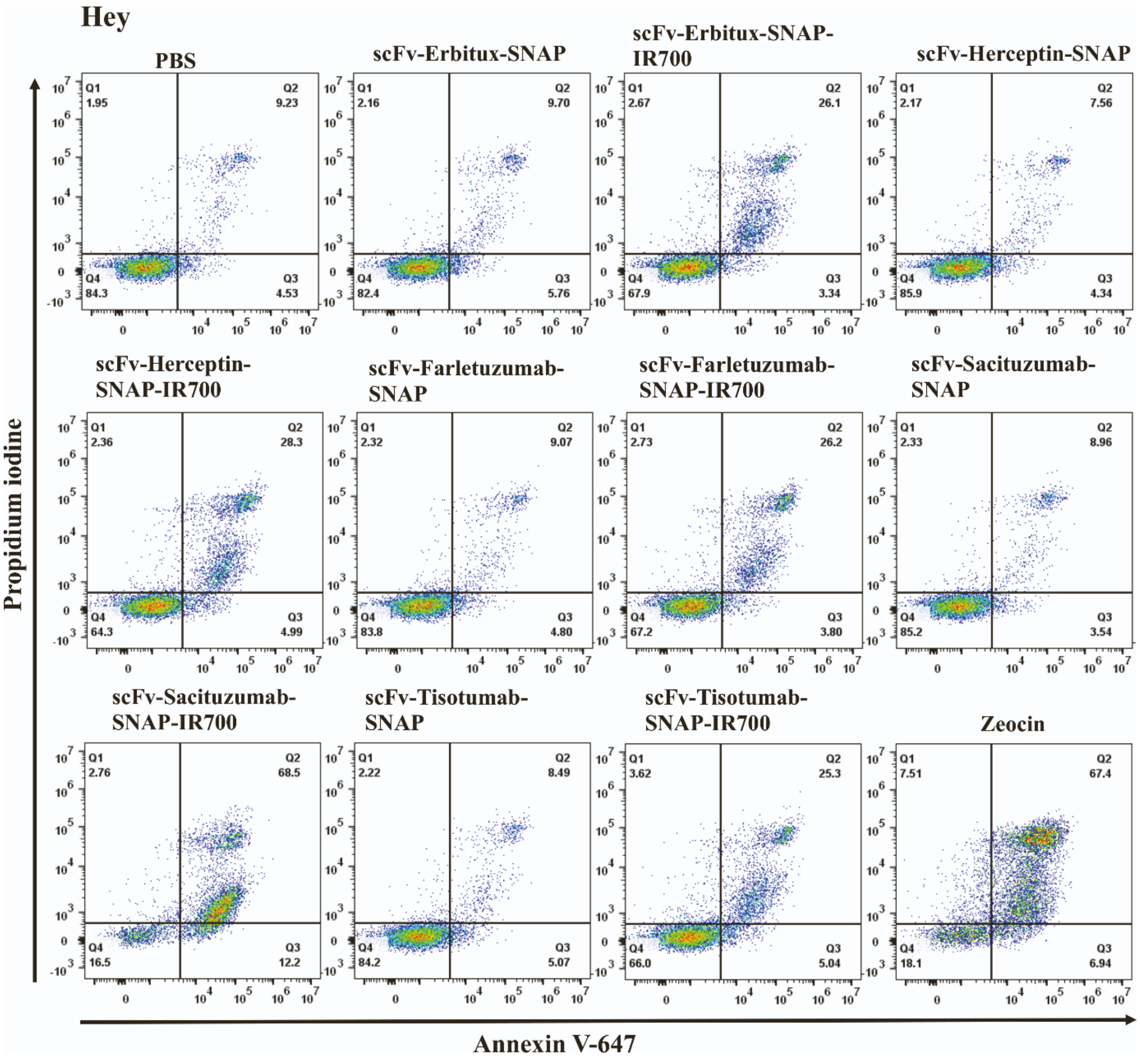

Figure S6: NIR-PIT agents mediated cell death in Hey cells. Cell death was measured by annexin assay after 24 h treatment. The flow cytometric dot plots depicted the living (annexin V⁻/PI⁻, Q4), early apoptotic (annexin V^+/PI^-, Q3), late apoptotic/necroptotic (annexin V^+/PI^+, Q2) and necrotic (annexin V^-/PI^+, Q1) cells. Cells treated with unconjugated scFv-SNAP localized mostly to the bottom left quadrant (Q4, viable cells) and NIR-PIT agents treated cells localized mostly to the top right quadrant (Q2, Necroptotic/Late apoptotic cells).

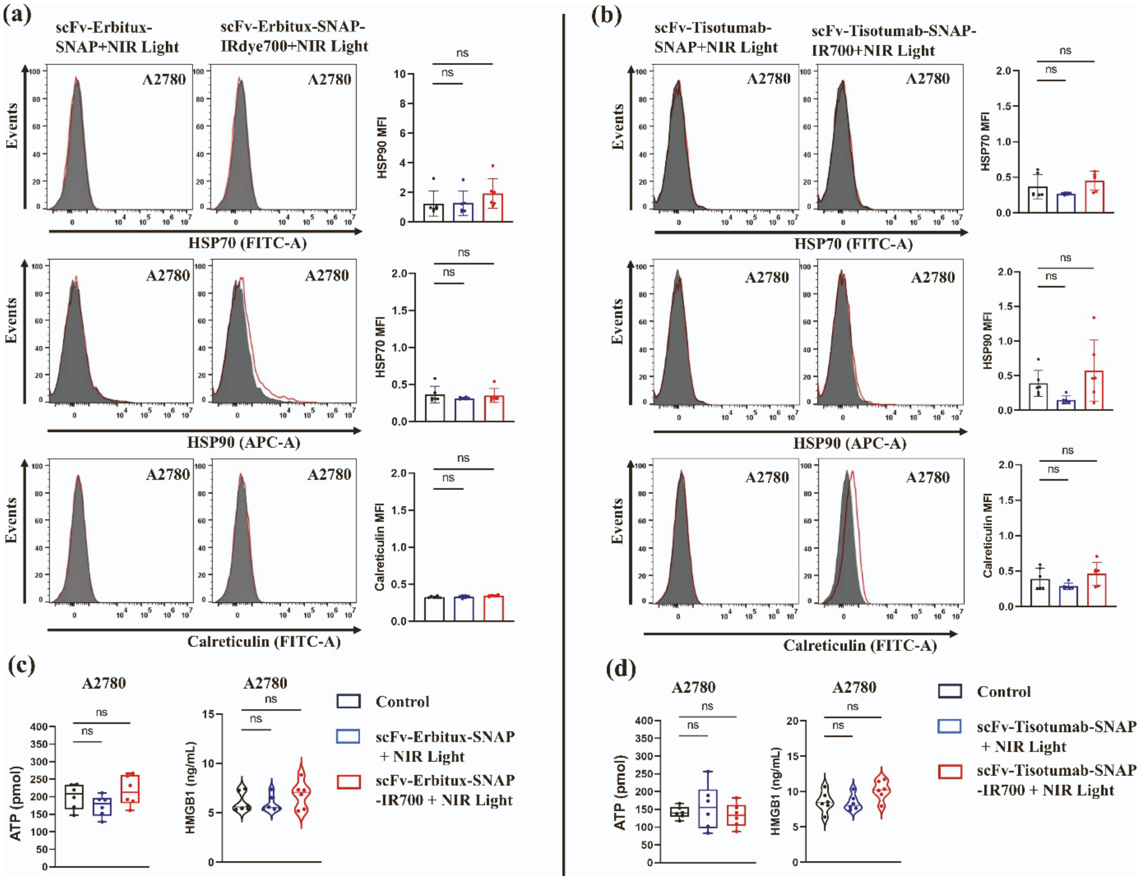

Figure S7: ICD marker analysis in A2780 cells after scFv-Erbitux-SNAP-IR700 and scFv-Tisotumab-SNAP-IR700 treatment. (a) The flow cytometric histograms represent the cell surface expression of calreticulin (red), HSP70 (red), and HSP90 (red) and the control (filled grey) in scFv-Erbitux-SNAP-IR700 and (b) scFv-Tisotumab-SNAP-IR700 treated A2780 cells. Only living cells were used in the analysis. MFI of cell surface calreticulin, HSP70, and HSP90 data are shown in bar graph with error bar (mean ± SD) (n=6). (c) Extracellular ATP and HMGB1 analysis in A2780 cells after scFv-Erbitux-SNAP-IR700 and (d) scFv-Tisotumab-SNAP-IR700 treatment. After 24 h NIR light irradiation, extracellular ATP was measured by ATP luminescence assay and extracellular HMGB1 was measured by ELISA assay. Data are presented in box plot (ATP release) and violin plot (HMGB1 release) as mean ± SD (n=6). Statistical significance was determined by a one-way ANOVA and Dunnett´s test. ns; non-significant.

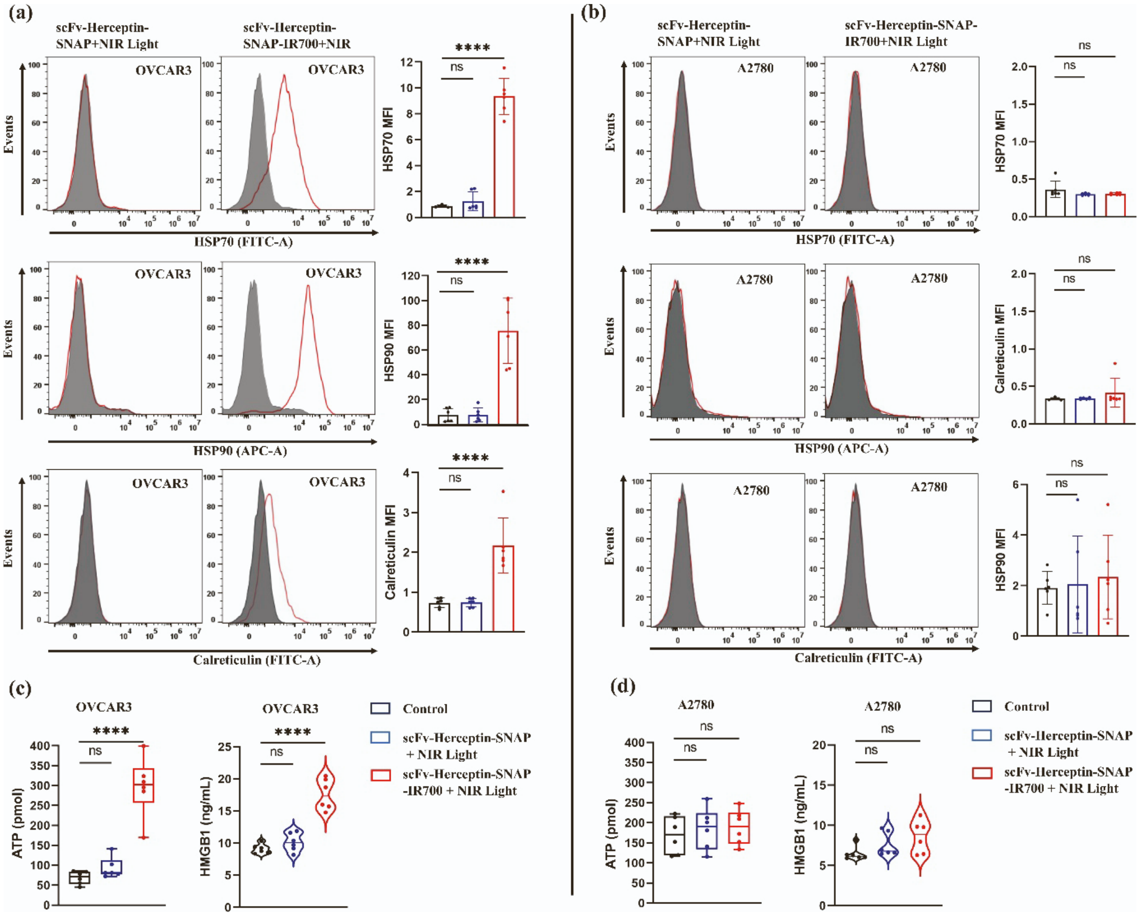

Figure S8: ICD marker analysis in OVCAR3 and A2780 cells after scFv-Herceptin-SNAP-IR700 treatment. (a) The flow cytometric histograms represent the cell surface expression of calreticulin (red), HSP70 (red), and HSP90 (red) and the control (filled grey) in scFv-Herceptin-SNAP-IR700 treated OVCAR3 cells and (b) A2780 cells. Only living cells were used in the analysis. MFI of cell surface calreticulin, HSP70, and HSP90 data are shown in bar graph with error bar (mean ± SD) (n=6). (c) Extracellular ATP and HMGB1 analysis in OVCAR3 and (d) A2780 cells after scFv-Herceptin-SNAP-IR700 treatment. After 24 h NIR light irradiation, extracellular ATP was measured by ATP luminescence assay and extracellular HMGB1 was measured by ELISA assay. Data are presented in box plot (ATP release) and violin plot (HMGB1 release) as mean ± SD (n=6). Statistical significance was determined by a one-way ANOVA and Dunnett´s test. ns; non-significant, ****$p \leq 0.0001$.

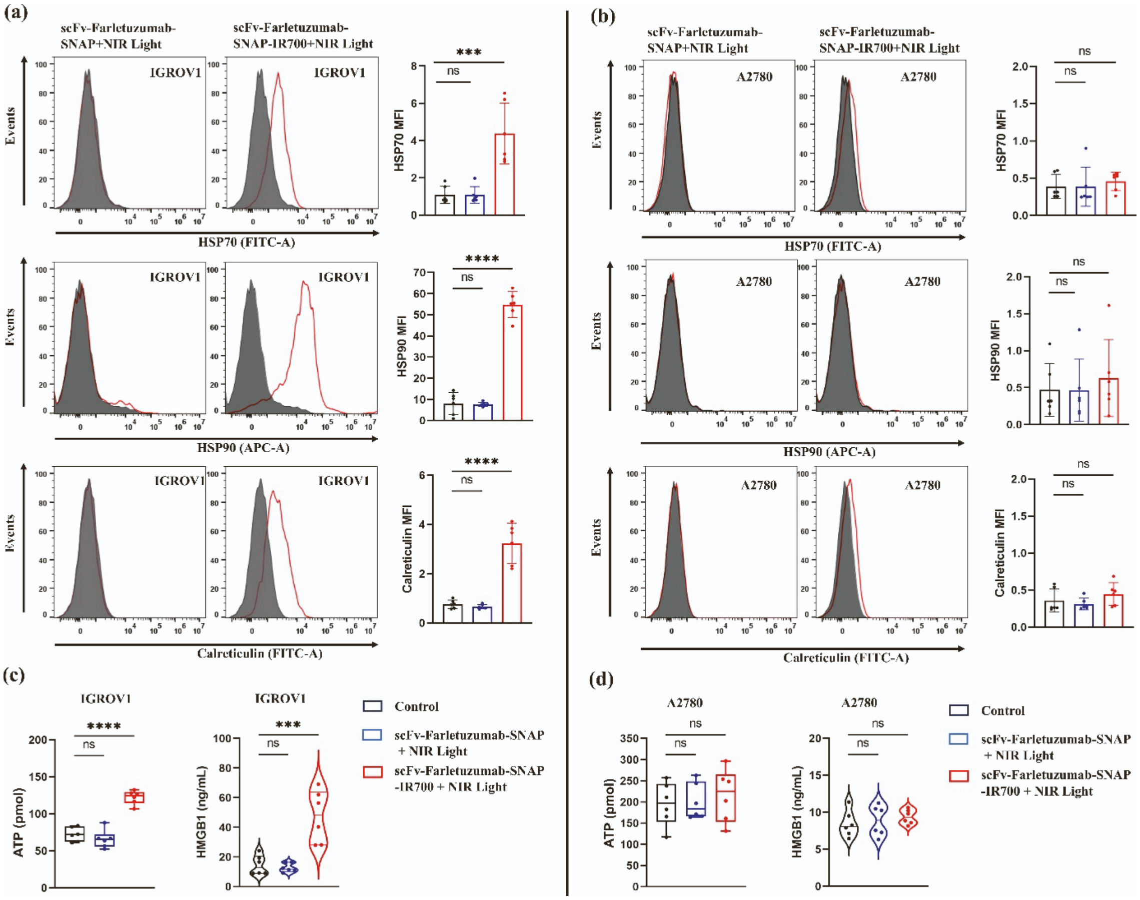

Figure S9: ICD marker analysis in IGROV1 and A2780 cells after scFv-Farletuzumab-SNAP-IR700 treatment. (a) The flow cytometric histograms represent the cell surface expression of calreticulin (red), HSP70 (red), and HSP90 (red) and the control (filled grey) in scFv-Farletuzumab-SNAP-IR700 treated IGROV1 cells and (b) A2780 cells. Only living cells were used in the analysis. MFI of cell surface calreticulin, HSP70, and HSP90 data are shown in bar graph with error bar (mean ± SD) (n=6). (c) Extracellular ATP and HMGB1 analysis in IGROV1 and (d) A2780 cells after scFv-Farletuzumab-SNAP-IR700 treatment. After 24 h NIR light irradiation, extracellular ATP was measured by ATP luminescence assay and extracellular HMGB1 was measured by ELISA assay. Data are presented in box plot (ATP release) and violin plot (HMGB1 release) as mean ± SD (n=6). Statistical significance was determined by a one-way ANOVA and Dunnett´s test. ns; non-significant, ***$p \leq 0.001$, ****$p \leq 0.0001$.

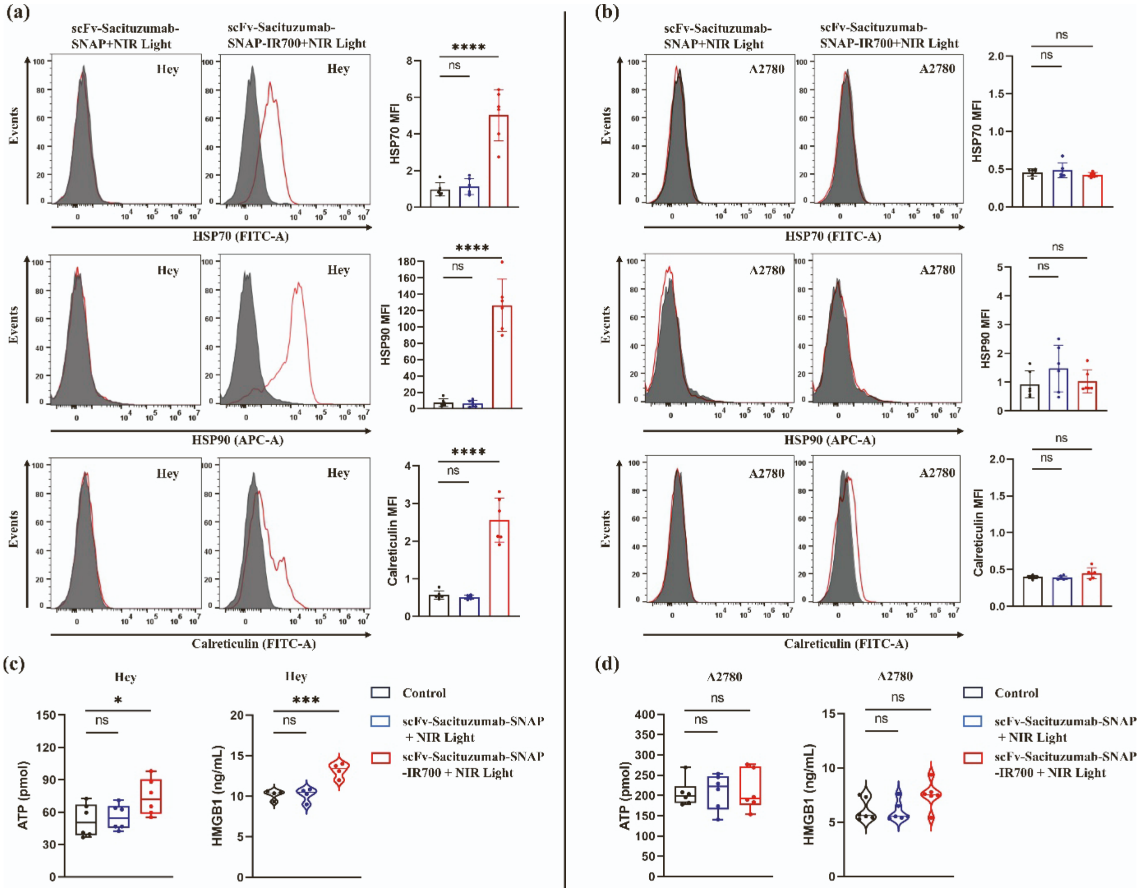

Figure S10: ICD marker analysis in Hey and A2780 cells after scFv-Sacituzumab-SNAP-IR700 treatment. (a) The flow cytometric histograms represent the cell surface expression of calreticulin (red), HSP70 (red), and HSP90 (red) and the control (filled grey) in scFv-Sacituzumab-SNAP-IR700 treated Hey cells and (b) A2780 cells. Only living cells were used in the analysis. MFI of cell surface calreticulin, HSP70, and HSP90 data are shown in bar graph with error bar (mean ± SD) (n=6). (c) Extracellular ATP and HMGB1 analysis in Hey and (d) A2780 cells after scFv-Sacituzumab-SNAP-IR700 treatment. After 24 h NIR light irradiation, extracellular ATP was measured by ATP luminescence assay and extracellular HMGB1 was measured by ELISA assay. Data are presented in box plot (ATP release) and violin plot (HMGB1 release) as mean ± SD (n=6). Statistical significance was determined by a one-way ANOVA and Dunnett´s test. ns; non-significant, *p ≤ 0.05, ***p ≤ 0.001, ****p ≤ 0.0001.

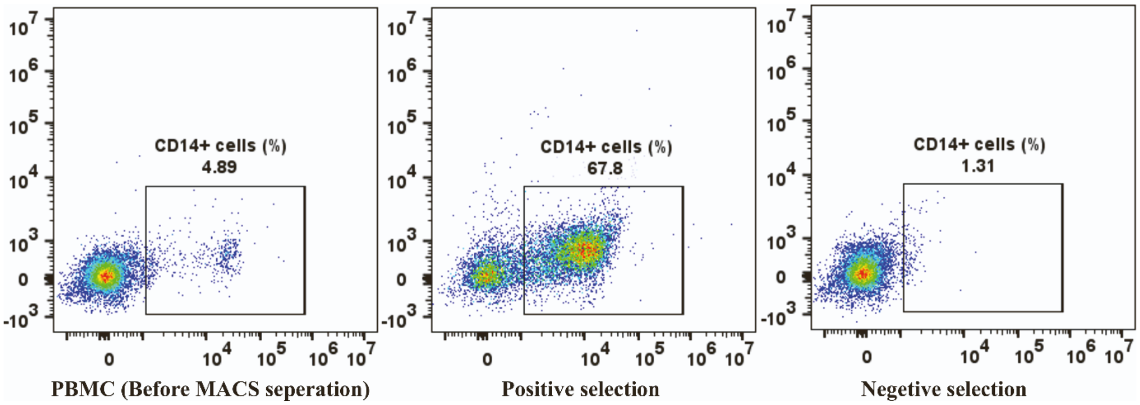

Figure S11: Isolation of CD14^{+} monocytes cells from PBMC using by positive selection using CD14 microbeads and MACS LS column. The efficiency of CD14^{+} monocytes cells isolation was investigated by staining with CD14 antibody using flow cytometry. The gated dot plot represents the percentage of CD14^{+} cells before the MACS separation (left dot plot), positive (middle dot plot) and negative (middle dot plot) selected cells after MACS separation.

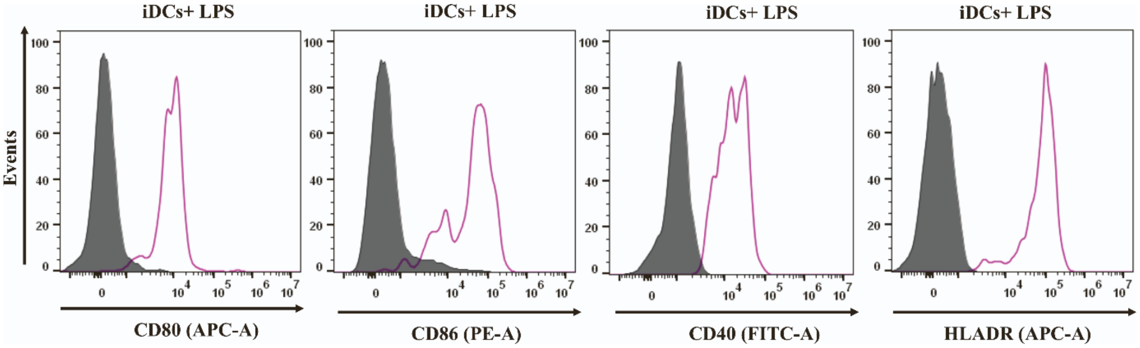

Figure S12: LPS treated iDCs promotes DCs maturation. Immature DCs were cultured with LPS. After 12h, the expressions of CD80, CD86, CD40 and HLADR on DCs were analyzed by flow cytometry. Immature DCs without co-culture are shown as a control. The expressions of CD80, CD86, HLADR, and CD40 on DCs are presented as flow cytometric histograms. The grey lines represent iDCs without LPS, the purple lines depict iDCs with LPS.

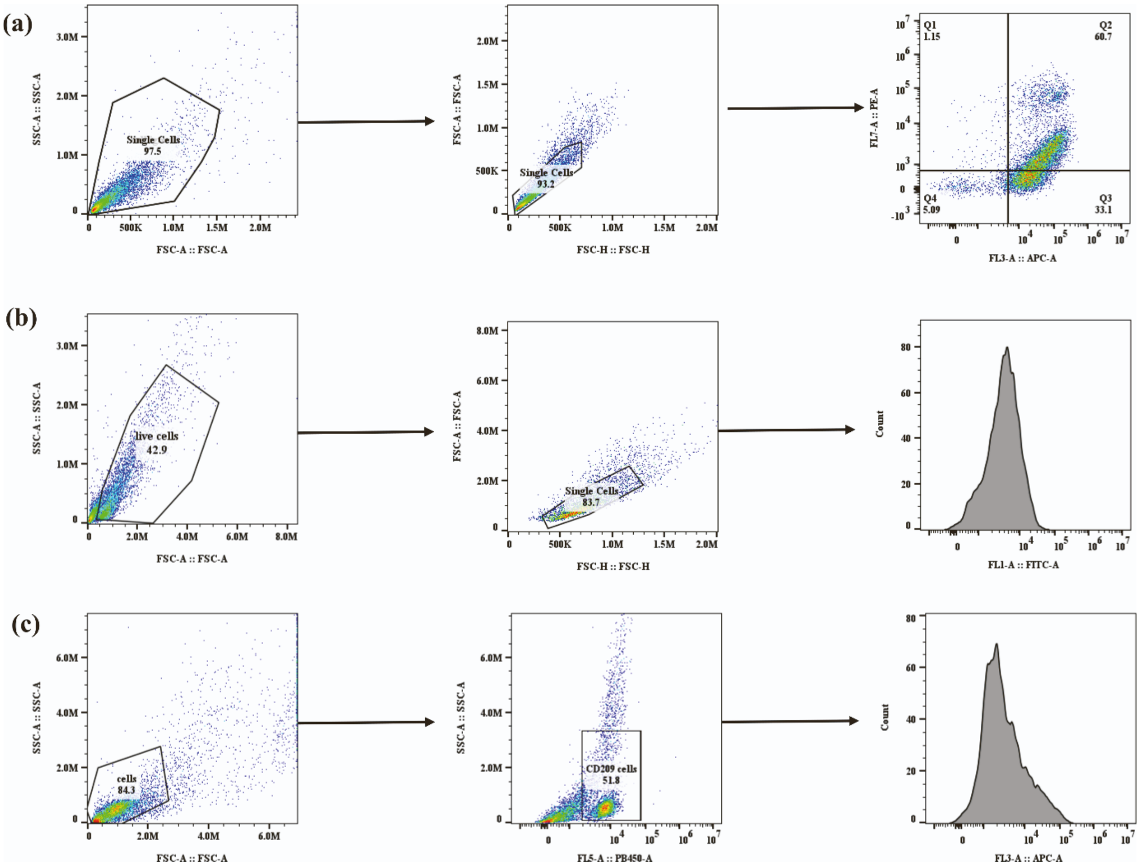

Figure S13: Gating strategy. (a) Gating strategy for annexin assay. (b) Gating strategy for ICD assay (calreticulin, HSP70, HSP90). (b) Gating strategy for DC maturation assay (CD80, CD86, CD40 and HLADR).
